# Physical, cognitive, and mental health impacts of COVID-19 after hospitalisation (PHOSP-COVID): a UK multicentre, prospective cohort study

**DOI:** 10.1016/S2213-2600(21)00383-0

**Published:** 2021-11

**Authors:** Rachael A Evans, Hamish McAuley, Ewen M Harrison, Aarti Shikotra, Amisha Singapuri, Marco Sereno, Omer Elneima, Annemarie B Docherty, Nazir I Lone, Olivia C Leavy, Luke Daines, J Kenneth Baillie, Jeremy S Brown, Trudie Chalder, Anthony De Soyza, Nawar Diar Bakerly, Nicholas Easom, John R Geddes, Neil J Greening, Nick Hart, Liam G Heaney, Simon Heller, Luke Howard, John R Hurst, Joseph Jacob, R Gisli Jenkins, Caroline Jolley, Steven Kerr, Onn M Kon, Keir Lewis, Janet M Lord, Gerry P McCann, Stefan Neubauer, Peter J M Openshaw, Dhruv Parekh, Paul Pfeffer, Najib M Rahman, Betty Raman, Matthew Richardson, Matthew Rowland, Malcolm G Semple, Ajay M Shah, Sally J Singh, Aziz Sheikh, David Thomas, Mark Toshner, James D Chalmers, Ling-Pei Ho, Alex Horsley, Michael Marks, Krisnah Poinasamy, Louise V Wain, Christopher E Brightling, PHOSP-COVID Collaborative Group, K Abel, H Adamali, D Adeloye, O Adeyemi, F Adeyemi, S Ahmad, R Ahmed, M Ainsworth, A Alamoudi, M Aljaroof, L Allan, R Allen, A Alli, B Al-Sheklly, D Altmann, D Anderson, M Andrews, A Angyal, C Antoniades, G Arbane, C Armour, N Armstrong, L Armstrong, H Arnold, D Arnold, M Ashworth, A Ashworth, H Assefa-Kebede, P Atkin, H Atkins, A Atkins, R Aul, C Avram, R Baggott, D Baguley, J K Baillie, S Bain, M Bakali, M Bakau, E Baldry, D Baldwin, C Ballard, J Bambrough, R E Barker, S Barratt, F Barrett, N Basu, R Batterham, H Baxendale, H Bayes, M Bayley, M Beadsworth, P Beirne, R Bell, D Bell, C Berry, S Betts, K Bhui, L Bishop, J Blaikely, C Bloomfield, A Bloss, A Bolger, C E Bolton, J Bonnington, A Botkai, M Bourne, C Bourne, E Bradley, K Bramham, L Brear, G Breen, J Breeze, A Briggs, E Bright, C E Brightling, S Brill, K Brindle, L Broad, M Broome, J S Brown, M Brown, J Brown, J Brown, R Brown, V Brown, A Brown, M Brown, A Brown, T Brugha, N Brunskill, M Buch, A Bularga, E Bullmore, D Burn, G Burns, J Busby, A Buttress, S Byrne, P Cairns, P C Calder, E Calvelo, B Card, L Carr, G Carson, P Carter, J Cavanagh, T Chalder, J D Chalmers, R C Chambers, K Channon, K Chapman, A Charalambou, N Chaudhuri, A Checkley, J Chen, L Chetham, E R Chilvers, H Chinoy, K Chong-James, N Choudhury, G Choudhury, P Chowdhury, P Chowienczyk, C Christie, D Clark, C Clark, J Clarke, P Clift, S Clohisey, Z Coburn, J Cole, C Coleman, D Connell, B Connolly, L Connor, A Cook, B Cooper, C Coupland, T Craig, P Crisp, D Cristiano, M G Crooks, A Cross, I Cruz, P Cullinan, L Daines, M Dalton, P Dark, J Dasgin, A David, C David, M Davies, G Davies, K Davies, F Davies, G A Davies, E Daynes, T De Silva, A De Soyza, B Deakin, A Deans, S Defres, A Dell, K Dempsey, J Dennis, A Dewar, R Dharmagunawardena, N Diar Bakerly, A Dipper, S Diver, S N Diwanji, M Dixon, R Djukanovic, H Dobson, C Dobson, S L Dobson, A B Docherty, A Donaldson, T Dong, N Dormand, A Dougherty, R Dowling, S Drain, P Dulawan, S Dunn, S Dunn, N Easom, C Echevarria, S Edwards, C Edwardson, B Elliott, A Elliott, Y Ellis, A Elmer, O Elneima, R A Evans, J Evans, H Evans, D Evans, R I Evans, R Evans, T Evans, L Fabbri, S Fairbairn, A Fairman, K Fallon, D Faluyi, C Favager, T Felton, J Finch, S Finney, H Fisher, S Fletcher, R Flockton, D Foote, A Ford, D Forton, R Francis, S Francis, C Francis, A Frankel, E Fraser, R Free, N French, J Fuld, J Furniss, L Garner, N Gautam, J R Geddes, P M George, J George, M Gibbons, L Gilmour, F Gleeson, J Glossop, S Glover, N Goodman, B Gooptu, T Gorsuch, E Gourlay, P Greenhaff, W Greenhalf, A Greenhalgh, N J Greening, J Greenwood, S Greenwood, R Gregory, D Grieve, M Gummadi, A Gupta, S Gurram, E Guthrie, K Hadley, A Haggar, K Hainey, P Haldar, I Hall, L Hall, M Halling-Brown, R Hamil, N A Hanley, H Hardwick, E Hardy, B Hargadon, K Harrington, V Harris, E M Harrison, P Harrison, N Hart, A Harvey, M Harvey, M Harvie, M Havinden-Williams, J Hawkes, N Hawkings, J Haworth, A Hayday, L G Heaney, J L Heeney, M Heightman, S Heller, M Henderson, L Hesselden, T Hillman, A Hingorani, T Hiwot, L P Ho, A Hoare, M Hoare, P Hogarth, A Holbourn, L Holdsworth, D Holgate, K Holmes, B Holroyd-Hind, A Horsley, A Hosseini, M Hotopf, L Houchen, L Howard, L Howard, A Howell, E Hufton, A Hughes, J Hughes, R Hughes, A Humphries, N Huneke, J R Hurst, R Hurst, M Husain, T Hussell, W Ibrahim, A Ient, L Ingram, K Ismail, T Jackson, J Jacob, W Y James, S Janes, H Jarvis, B Jayaraman, R G Jenkins, P Jezzard, K Jiwa, S Johnson, C Johnson, D Johnston, C Jolley, C J Jolley, I Jones, S Jones, D Jones, H Jones, G Jones, M Jones, S Jose, T Kabir, G Kaltsakas, V Kamwa, P Kar, Z Kausar, S Kelly, S Kerr, A L Key, F Khan, K Khunti, C King, B King, P Kitterick, P Klenerman, L Knibbs, S Knight, A Knighton, O M Kon, S Kon, S S Kon, A Korszun, C Kotanidis, I Koychev, P Kurupati, J Kwan, C Laing, H Lamlum, G Landers, C Langenberg, D Lasserson, A Lawrie, A Lea, O C Leavy, D Lee, E Lee, K Leitch, R Lenagh, K Lewis, V Lewis, K E Lewis, J Lewis, N Lewis-Burke, T Light, L Lightstone, L Lim, S Linford, A Lingford-Hughes, M Lipman, K Liyanage, A Lloyd, S Logan, D Lomas, N I Lone, R Loosley, J M Lord, H Lota, A Lucey, G MacGowan, I Macharia, C Mackay, L Macliver, S Madathil, G Madzamba, N Magee, N Mairs, N Majeed, E Major, M Malim, G Mallison, W Man, S Mandal, K Mangion, P Mansoori, S Marciniak, M Mariveles, M Marks, B Marshall, A Martineau, N Maskell, D Matila, L Matthews, J Mayet, S McAdoo, H McAllister-Williams, P McArdle, A McArdle, D McAulay, H McAuley, D F McAuley, K McCafferty, G P McCann, H McCauley, P McCourt, L Mcgarvey, J McGinness, A McGovern, H McGuinness, I B McInnes, K McIvor, E McIvor, A McMahon, M J McMahon, L McMorrow, T Mcnally, M McNarry, A McQueen, H McShane, S Megson, J Meiring, D Menzies, A Michael, L Milligan, N Mills, J Mitchell, A Mohamed, P L Molyneaux, W Monteiro, A Morley, L Morrison, R Morriss, A Morrow, A Moss, A J Moss, P Moss, E Mukaetova-Ladinska, U Munawar, E Murali, J Murira, H Nassa, P Neill, S Neubauer, D Newby, H Newell, A Newton Cox, T Nicholson, D Nicoll, C M Nolan, M J Noonan, P Novotny, J Nunag, J Nyaboko, L O'Brien, N Odell, G Ogg, O Olaosebikan, C Oliver, Z Omar, P J M Openshaw, P Rivera-Ortega, R Osbourne, M Ostermann, C Overton, J Oxton, E Pacpaco, S Paddick, P Papineni, K Paradowski, M Pareek, D Parekh, H Parfrey, C Pariante, S Parker, M Parkes, J Parmar, R Parvin, S Patale, B Patel, S Patel, M Patel, B Pathmanathan, M Pavlides, J E Pearl, D Peckham, J Pendlebury, Y Peng, C Pennington, I Peralta, E Perkins, T Peto, N Petousi, J Petrie, P Pfeffer, J Phipps, J Pimm, K Piper Hanley, R Pius, S Plein, T Plekhanova, K Poinasamy, O Polgar, L Poll, J C Porter, S Portukhay, N Powell, L Price, D Price, A Price, C Price, A Prickett, S Quaid, J Quigley, J Quint, H Qureshi, N Rahman, M Rahman, M Ralser, B Raman, A Ramos, J Rangeley, T Rees, K Regan, A Richards, M Richardson, E Robertson, J Rodgers, G Ross, J Rossdale, A Rostron, A Routen, A Rowland, M J Rowland, J Rowland, S L Rowland-Jones, K Roy, I Rudan, R Russell, E Russell, R Sabit, E K Sage, N Samani, R Samuel, E Sapey, D Saralaya, A Saratzis, J Sargeant, T Sass, N Sattar, K Saunders, R Saunders, W Saxon, A Sayer, W Schwaeble, J Scott, K Scott, N Selby, M G Semple, M Sereno, K Shah, A Shah, P Shah, M Sharma, M Sharpe, C Sharpe, V Shaw, A Sheikh, K Shevket, A Shikotra, J Short, S Siddiqui, L Sigfrid, G Simons, J Simpson, A Singapuri, S J Singh, C Singh, S Singh, J Skeemer, I Smith, J Smith, L Smith, A Smith, M Soares, D Southern, M Spears, L G Spencer, F Speranza, L Stadon, S Stanel, M Steiner, D Stensel, M Stern, I Stewart, J Stockley, R Stone, A Storrie, K Storton, E Stringer, C Subbe, C Sudlow, Z Suleiman, C Summers, C Summersgill, D Sutherland, D L Sykes, R Sykes, N Talbot, A L Tan, C Taylor, A Taylor, A Te, H Tedd, C J Tee, H Tench, S Terry, S Thackray-Nocera, F Thaivalappil, D Thickett, D Thomas, D C Thomas, A K Thomas, A A R Thompson, T Thompson, T Thornton, R S Thwaites, M Tobin, G F Toingson, C Tong, M Toshner, R Touyz, K A Tripp, E Tunnicliffe, E Turner, L Turtle, H Turton, R Ugwuoke, R Upthegrove, J Valabhji, K Vellore, E Wade, L V Wain, L O Wajero, S Walder, S Walker, E Wall, T Wallis, S Walmsley, S Walsh, J A Walsh, L Watson, J Watson, L Watson, E Watson, C Welch, H Welch, B Welsh, S Wessely, S West, H Wheeler, V Whitehead, J Whitney, S Whittaker, B Whittam, J Wild, M Wilkins, D Wilkinson, N Williams, B Williams, J Williams, S A Williams-Howard, M Willicombe, G Willis, D Wilson, I Wilson, N Window, M Witham, R Wolf-Roberts, F Woodhead, J Woods, D Wootton, J Worsley, D Wraith, L Wright, C Wright, S Wright, C Xie, S Yasmin, T Yates, K P Yip, B Young, S Young, A Young, A J Yousuf, A Yousuf, A Zawia, B Zhao, O Zongo

**Affiliations:** a Institute for Lung Health, Leicester NIHR Biomedical Research Centre, University of Leicester, Leicester, UK; b Department of Health Sciences, University of Leicester, Leicester, UK; c Department of Cardiovascular Sciences, University of Leicester, Leicester, UK; d Usher Institute, University of Edinburgh, Edinburgh, UK; e Roslin Institute, University of Edinburgh, Edinburgh, UK; f Royal Infirmary of Edinburgh, NHS Lothian, Edinburgh, UK; g UCL Respiratory, Department of Medicine, University College London, London, UK; h Centre for Medical Image Computing, University College London, London, UK; i Lungs for Living Research Centre, University College London, London, UK; j Department of Psychological Medicine, Institute of Psychiatry, Psychology and Neuroscience, King's College London, London, UK; k Centre for Human and Applied Physiological Sciences, School of Basic and Medical Biosciences, Faculty of Life Sciences and Medicine, King's College London, London, UK; l Population Health Sciences Institute, Newcastle University, Newcastle upon Tyne, UK; m Newcastle upon Tyne Teaching Hospitals Trust, Newcastle upon Tyne, UK; n Manchester Metropolitan University, Manchester, UK; o Salford Royal NHS Foundation Trust, Manchester, UK; p Infection Research Group, Hull University Teaching Hospitals, Hull, UK; q NIHR Oxford Health Biomedical Research Centre, University of Oxford, Oxford, UK; r Division of Cardiovascular Medicine, Radcliffe Department of Medicine, University of Oxford, Oxford, UK; s Oxford Respiratory Trials Unit, University of Oxford, Oxford, UK; t Kadoorie Centre for Critical Care Research, Nuffield Department of Clinical Neurosciences, University of Oxford, Oxford, UK; u MRC Human Immunology Unit, University of Oxford, Oxford, UK; v Oxford Health NHS Foundation Trust, Oxford, UK; w Lane Fox Respiratory Service, Guy's and St Thomas' NHS Foundation Trust, London, UK; x Wellcome-Wolfson Institute for Experimental Medicine, Queen's University Belfast, Belfast, UK; y Belfast Health & Social Care Trust, Belfast, UK; z Department of Oncology and Metabolism, University of Sheffield, Sheffield, UK; aa Imperial College Healthcare NHS Trust, London, UK, University College London, London, UK; ab National Heart and Lung Institute, Imperial College London, London, UK; ac Immunology and Inflammation, Imperial College London, London, UK; ad Hywel Dda University Health Board, Wales, UK; ae University of Swansea, Swansea, UK; af Respiratory Innovation Wales, Llanelli, UK; ag Institute of Inflammation and Ageing, University of Birmingham, Birmingham, UK; ah Leicester NIHR Biomedical Research Centre, University of Leicester, Leicester, UK; ai Department of Acute Medicine, Queen Elizabeth Hospital Birmingham, Birmingham, UK; aj Barts Health NHS Trust, London, UK; ak Queen Mary University of London, London, UK; al NIHR Health Protection Research Unit in Emerging and Zoonotic Infections, Institute of Infection, Veterinary and Ecological Sciences, University of Liverpool, UK; am Respiratory Medicine, Alder Hey Children's Hospital, Liverpool, UK; an King's College London British Heart Foundation Centre and King's College Hospital NHS Foundation Trust, London, UK; ao Cambridge NIHR Biomedical Research Centre, Cambridge, UK; ap NIHR Cambridge Clinical Research Facility, Cambridge, UK; aq University of Dundee, Ninewells Hospital and Medical School, Dundee, UK; ar Division of Infection, Immunity & Respiratory Medicine, Faculty of Biology, Medicine and Health, University of Manchester, Manchester, UK; as Department of Clinical Research, London School of Hygiene & Tropical Medicine, London, UK; at Hospital for Tropical Diseases, University College London Hospital, London, UK; au Asthma UK and British Lung Foundation, London, UK

## Abstract

**Background:**

The impact of COVID-19 on physical and mental health and employment after hospitalisation with acute disease is not well understood. The aim of this study was to determine the effects of COVID-19-related hospitalisation on health and employment, to identify factors associated with recovery, and to describe recovery phenotypes.

**Methods:**

The Post-hospitalisation COVID-19 study (PHOSP-COVID) is a multicentre, long-term follow-up study of adults (aged ≥18 years) discharged from hospital in the UK with a clinical diagnosis of COVID-19, involving an assessment between 2 and 7 months after discharge, including detailed recording of symptoms, and physiological and biochemical testing. Multivariable logistic regression was done for the primary outcome of patient-perceived recovery, with age, sex, ethnicity, body-mass index, comorbidities, and severity of acute illness as covariates. A post-hoc cluster analysis of outcomes for breathlessness, fatigue, mental health, cognitive impairment, and physical performance was done using the clustering large applications k-medoids approach. The study is registered on the ISRCTN Registry (ISRCTN10980107).

**Findings:**

We report findings for 1077 patients discharged from hospital between March 5 and Nov 30, 2020, who underwent assessment at a median of 5·9 months (IQR 4·9–6·5) after discharge. Participants had a mean age of 58 years (SD 13); 384 (36%) were female, 710 (69%) were of white ethnicity, 288 (27%) had received mechanical ventilation, and 540 (50%) had at least two comorbidities. At follow-up, only 239 (29%) of 830 participants felt fully recovered, 158 (20%) of 806 had a new disability (assessed by the Washington Group Short Set on Functioning), and 124 (19%) of 641 experienced a health-related change in occupation. Factors associated with not recovering were female sex, middle age (40–59 years), two or more comorbidities, and more severe acute illness. The magnitude of the persistent health burden was substantial but only weakly associated with the severity of acute illness. Four clusters were identified with different severities of mental and physical health impairment (n=767): very severe (131 patients, 17%), severe (159, 21%), moderate along with cognitive impairment (127, 17%), and mild (350, 46%). Of the outcomes used in the cluster analysis, all were closely related except for cognitive impairment. Three (3%) of 113 patients in the very severe cluster, nine (7%) of 129 in the severe cluster, 36 (36%) of 99 in the moderate cluster, and 114 (43%) of 267 in the mild cluster reported feeling fully recovered. Persistently elevated serum C-reactive protein was positively associated with cluster severity.

**Interpretation:**

We identified factors related to not recovering after hospital admission with COVID-19 at 6 months after discharge (eg, female sex, middle age, two or more comorbidities, and more acute severe illness), and four different recovery phenotypes. The severity of physical and mental health impairments were closely related, whereas cognitive health impairments were independent. In clinical care, a proactive approach is needed across the acute severity spectrum, with interdisciplinary working, wide access to COVID-19 holistic clinical services, and the potential to stratify care.

**Funding:**

UK Research and Innovation and National Institute for Health Research.

## Introduction

As of September, 2021, the number of reported cases of COVID-19 exceeds 225 million worldwide, with more than 4·6 million deaths. Of the 7·4 million UK cases, 536 000 have been admitted to hospital.^1^ Over the course of the pandemic, in-hospital mortality has reduced from more than 30% initially to less than 20% currently,^2^ leaving more than 300 000 post-hospitalisation survivors of COVID-19 in the UK. It is well established that in survivorship cohorts of hospitalised patients following critical illness, prolonged morbidity with reduced functional status and impaired mental health persists for many years.^3^

**Research in context**

**Evidence before this study**

We searched PubMed for studies of the long-term effects of COVID-19 on individuals after hospitalisation, published up to March 22, 2021. We used the search terms (“COVID-19” and “hospital” and [“long-term” OR “sequelae” OR “consequences”] and “cohort”). We excluded studies that had less than 3 months of follow-up after hospital discharge, those with fewer than 500 participants, and those that considered only single-organ or system effects. The impact of COVID-19 on physical and mental health and employment after hospitalisation is not well understood. A large, single-centre study from Wuhan, China, highlighted the burden of disease persisting at 6 months, with 63% experiencing persistent fatigue and 23% experiencing symptoms of anxiety and depression. Much remains unknown about the characteristics of those experiencing a prolonged recovery.

**Added value of this study**

To our knowledge, we report the first and largest multicentre study, involving a diverse cohort in terms of ethnicity and spectrum of acute illness severity. Participants were prospectively recruited and attended an in-person research visit to assess their physical, mental, and cognitive status at a median of 6 months after hospital discharge, including comparisons with pre-COVID-19 status. Our findings confirm a large burden of symptoms persisting at 5 months after discharge, but also highlight a substantial proportion of survivors reporting a new disability and disruption to employment. Predictors of not recovering included female sex, comorbidities, middle age, and requiring invasive mechanical ventilation during admission. The mental and physical health impairments were only weakly associated with the severity of acute illness. We progress the understanding of the burden of disease after hospitalisation from COVID-19 by describing four clusters of recovery phenotype, in terms of mental health, physical performance, and cognition. There were significant differences in perceived recovery, impact on health-related quality of life, occupation change due to health, and disability across the four clusters, and higher C-reactive protein levels among patients experiencing the most severe ongoing impairments.

**Implications of all the available evidence**

Our findings suggest that there are underlying mechanisms causing severe mental and physical impairments, independent of the degree of acute lung injury and potentially related to persisting systemic inflammation. Our data, along with previous reports, suggest that a proactive approach and holistic clinical care are needed owing to the large burden of health impairments. The four clusters highlight the potential to stratify and personalise care, and emphasise the need for wide access to interventions to improve mental, physical, and cognitive health.

The largest post-hospitalisation cohort study of COVID-19 survivors published to date (from Wuhan, China) reported ongoing symptoms at 6 months with a positive association with severity of acute illness.^4^ However, even in the milder group (those not requiring supplemental oxygen), more than 80% had persistent symptoms at 6 months.^4^

In the UK, the Post-hospitalisation COVID-19 study (PHOSP-COVID) was established as a national consortium to understand and improve long-term health outcomes after COVID-19. In this first analysis, we report the outcomes at first review for patients hospitalised with COVID-19 (who were discharged between March and November, 2020). The aim was to determine the impact on health and employment, to identify factors associated with recovery, and to describe recovery phenotypes.

## Methods

### Study design and participants

This prospective, longitudinal cohort study recruited patients aged 18 years or older who were discharged from one of 53 National Health Service (NHS) hospitals between March 5 and Nov 30, 2020 across England, Northern Ireland, Scotland, and Wales after admission to a medical assessment or ward for confirmed or clinician-diagnosed COVID-19. We excluded patients who had a confirmed diagnosis of a pathogen unrelated to the objectives of this study, who attended an accident and emergency department but were not admitted, or who had another life-limiting illness with life expectancy of less than 6 months, such as disseminated malignancy. The PHOSP-COVID study includes collection of routine clinical data with linkage to retrospective and prospective health and social care records (Tier 1), enhanced clinical data collection and research-specific biosampling (Tier 2), and recall of participants by genotype and phenotype for more detailed studies (Tier 3). Participants from Tier 2 who have undergone careful phenotyping are included in this study. This exploratory analysis is restricted to participants who consented to attend two follow-up research visits within 1 year after discharge (Tier 2) in addition to routine clinical care. The current version of the study protocol is provided in appendix 2.

Written informed consent was obtained from all study participants. The study was approved by the Leeds West Research Ethics Committee (20/YH/0225) and is registered on the ISRCTN Registry (ISRCTN10980107).

### Procedures

Participants were invited to attend a research visit between 2 and 7 months (plus or minus 2 weeks) after hospital discharge. Where possible, this was scheduled alongside clinical follow-up, and data from clinically collected assessments within 4 weeks of a research visit were used. A core set of data variables for Tier 2 were obtained from the clinical records if part of clinical care or at the research visit (when not performed clinically; appendix 1 pp 10–13). The list of Tier 2 outcome measures is shown in appendix 1 (p 10), indicating which measures are included in this analysis. Baseline data

Patient demographics and characteristics of their acute COVID-19 admission, including results of a PCR test for SARS-CoV-2, treatments, and organ support received, were obtained from hospital notes by the study team at each site. The Index of Multiple Deprivation, a geographical measure of social deprivation, was obtained for each participant using their postcode.^5^ Severity of acute illness was determined by the highest level of organ support received while admitted, categorised using the WHO Clinical Progression Scale as class 3–4 (no continuous supplemental oxygen needed), class 5 (continuous supplemental oxygen only), class 6 (continuous positive airway pressure ventilation, bi-level positive airway pressure, or high-flow nasal oxygen), or class 7–9 (invasive mechanical ventilation or extracorporeal membrane oxygenation).^6^ For the purpose of this analysis, those receiving renal replacement therapy acutely were assigned to class 7–9. Only participants with complete data for date of admission, organ support during admission, sex at birth, and attendance at a research visit 2–7 months after discharge were included in this analysis.

### Outcomes

The primary outcome measures assessed health status and patient-perceived recovery. Patient-reported outcomes were collected using the following validated questionnaires: EuroQol five-dimension five-level (EQ-5D-5L) questionnaire, including the EuroQol Visual Analogue Scale (EQ-VAS),^7^ the Generalized Anxiety Disorder 7-item scale (GAD-7),^8^ the Patient Health Questionnaire-9 (PHQ-9),^9^ the Post Traumatic Stress Disorder Checklist (PCL-5),^10^ Dyspnoea-12,^11^ the Functional Assessment of Chronic Illness Therapy (FACIT) Fatigue Scale,^12^ the Brief Pain Inventory (BPI),^13^ and the Washington Group Short Set on Functioning (WG-SS; appendix 1 pp 11–13).^14^ In addition, participants completed a study-specific clinical questionnaire that asked about their general recovery, symptoms, and changes to their working status since their COVID-19 admission (appendix p 11). Patient recovery was assessed by asking the question “Do you feel fully recovered?” and patients could respond “Yes”, “No”, or “Unsure”. The Montreal Cognitive Assessment (MoCA),^15^ the Rockwood Clinical Frailty Scale,^16^ the incremental shuttle walk test (ISWT),^17^ the short physical performance battery (SPPB),^18^ and body-mass index (BMI) were measured (appendix 1 pp 11–13). In addition, pulmonary function tests and biochemical tests, including C-reactive protein, brain natriuretic peptide (BNP) or N-terminal-BNP (or both), and haematological and renal profiles, were done.

### Statistical analysis

Continuous variables were presented as median (IQR) or mean (SD). Binary and categorical variables were presented as counts and percentages. Participants were stratified by the severity of their acute COVID-19 illness (based on four independent categories defined by WHO), by number of pre-existing comorbidities, or by cluster (see methods below). Missing data were reported within each variable and per category. A χ^2^ test was used to identify differences in proportions across multiple categories. For normally distributed continuous data, analysis of variance (ANOVA F-test) was used to test differences across categories, with Kruskal-Wallis tests used for non-normally distributed data.

We reported univariable and multivariable logistic regression with and without imputed data where applicable. Missing values in variables were handled using multiple imputation by chained equations, under the missing-at-random assumption. Ten sets, each with ten iterations, were imputed using the following variables: age, sex, ethnicity, Index of Multiple Deprivation, BMI (measured at the 2–7 month follow-up visit, and used as a surrogate for BMI at admission), severity (WHO Clinical Progression Scale), comorbidity categories,^19^ admission duration, treatment with steroids, treatment with antibiotics, treatment with therapeutic anticoagulation, and the outcome variable (self-reported recovery from COVID-19). Further models were done, including discharge to review time and without imputation of the outcome variable. Model derivation and validation was done in imputed datasets, with Rubin's rules^20^ used to combine results. Patient-perceived recovery from COVID-19 was modelled using hierarchical multivariable logistic regression, with admission hospital incorporated as a random effect. Criterion-based model building used the following principles: relevant explanatory variables were identified a priori for exploration, interactions were checked at the first-order level and incorporated if significant, and the final model selection was informed by the Akaike Information Criterion and C-statistic, with appropriate assumptions checked, including the distribution of residuals. Sensitivity analyses were done using complete-case data. The final model was presented as a forest plot with odds ratios (ORs) and 95% CIs.

In a post-hoc analysis, unsupervised clustering of patient symptom questionnaire, physical performance, and cognitive assessment data (Dyspnoea-12, FACIT, GAD-7, PHQ-9, PCL-5, SPPB, and MoCA as continuous variables) was undertaken using the clustering large applications k-medoids approach.^21^ Scores were centred, normalised, and transformed so that higher burden of disease represented higher values. A Euclidean distance metric was used, and the optimal number of clusters was chosen using a silhouette plot. Cluster membership was determined for each individual and characteristics were presented as stratified tables. Pearson correlation was used for the comparison between BMI and C-reactive protein.

All tests were two-tailed and p values of less than 0·05 were considered statistically significant. We did not adjust for multiple testing. We used R (version 3.6.3) with the *finalfit, tidyverse, mice, cluster*, and *recipes* packages for all statistical analysis.

### Role of the funding source

The funder of the study had no role in study design, data collection, data analysis, data interpretation, or writing of the report.

## Results

**Figure 1:**
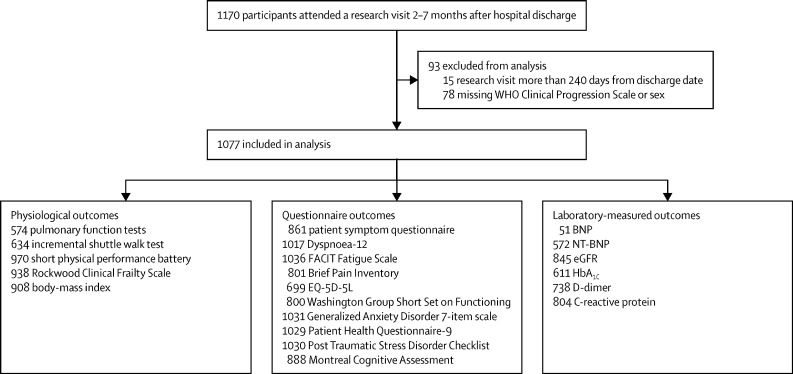
Flow diagram of participants BNP=brain natriuretic peptide. eGFR=estimated glomerular filtration rate. EQ-5D-5L=EuroQol five-dimension five-level questionnaire. FACIT=Functional Assessment of Chronic Illness Therapy. HbA_1c_=glycated haemoglobin. NT-BNP=N-terminal brain natriuretic peptide.

1170 patients who were discharged from hospital between March 5 and Nov 30, 2020, after treatment for COVID-19, were assessed between Aug 14, 2020, and March 5, 2021; 1077 of these patients were included in the analysis (figure 1). Overall, the majority of participants were male and of white ethnicity (table 1). 315 (29·2%) of 1077 had no comorbidities, 222 (20·6%) had one comorbidity, and 540 (50·1%) had at least two comorbidities. The most common comorbidities were cardiovascular, respiratory, and type 2 diabetes (table 1; a complete list of recorded comorbidities is shown in appendix 1, pp 14–15). The cohort demographics and pre-existing comorbidities were similar across the severity of acute illness categories, except for a higher proportion of males (73·6%) among those receiving mechanical ventilation (WHO class 7–9; table 1). Before their hospital admission, 641 (67·5%) of 950 participants were working either full-time (n=547) or part-time (n=94; appendix 1 p 16). The median length of stay was 9 days (IQR 4–21) and 894 (89·5%) of 999 patients had a positive PCR test for COVID-19 at the time of admission (table 1).

**Table 1:** Comparison of participant demographics, clinical characteristics, and admission characteristics stratified by acute illness severity

			**WHO class 3–4**	**WHO class 5**	**WHO class 6**	**WHO class 7–9**	**Total**
**Demographics**							
Participants			226 (21·0%)	378 (35·1%)	185 (17·2%)	288 (26·7%)	1077
Age at admission, years			54·8 (15·0)	60·7 (12·5)	59·1 (12·8)	55·7 (10·9)	57·9 (13·0)
Missing data			3	7	8	9	27
Sex at birth							
	Female		115 (50·9%)	134 (35·4%)	59 (31·9%)	76 (26·4%)	384 (35·7%)
	Male		111 (49·1%)	244 (64·6%)	126 (68·1%)	212 (73·6%)	693 (64·3%)
Ethnicity							
	White		148 (67·3%)	251 (70·3%)	130 (73·0%)	181 (64·6%)	710 (68·6%)
	South Asian		48 (21·8%)	49 (13·7%)	28 (15·7%)	41 (14·6%)	166 (16·0%)
	Black		18 (8·2%)	27 (7·6%)	13 (7·3%)	31 (11·1%)	89 (8·6%)
	Mixed		5 (2·3%)	12 (3·4%)	2 (1·1%)	4 (1·4%)	23 (2·2%)
	Other		1 (0·5%)	18 (5·0%)	5 (2·8%)	23 (8·2%)	47 (4·5%)
	Missing data		6	21	7	8	42
Occupation status							
	Working full time		110 (54·5%)	172 (52·6%)	90 (53·9%)	175 (68·9%)	547 (57·6%)
	Working part time		23 (11·4%)	31 (9·5%)	19 (11·4%)	21 (8·3%)	94 (9·9%)
	Other occupation status		69 (34·2%)	124 (37·9%)	58 (34·7%)	58 (22·8%)	309 (32·5%)
	Missing data		24	51	18	34	127
Health-care worker			52 (24·6%)	58 (18·2%)	30 (18·9%)	57 (21·3%)	197 (20·6%)
	Missing data		15	59	26	21	121
IMD							
	1 (most deprived)		44 (19·9%)	84 (22·6%)	30 (17·0%)	57 (20·0%)	215 (20·4%)
	2		43 (19·5%)	84 (22·6%)	51 (29·0%)	64 (22·5%)	242 (23·0%)
	3		45 (20·4%)	63 (17·0%)	34 (19·3%)	60 (21·1%)	202 (19·2%)
	4		47 (21·3%)	71 (19·1%)	30 (17·0%)	48 (16·8%)	196 (18·6%)
	5 (least deprived)		42 (19·0%)	69 (18·6%)	31 (17·6%)	56 (19·6%)	198 (18·8%)
	Missing data		5	7	9	3	24
**Clinical characteristics**							
BMI			29·1 (25·1–33·5)	29·8 (26·8–34·0)	32·1 (28·2–35·9)	30·3 (27·7–34·8)	30·1 (26·8–34·5)
	<30		103 (56·3%)	166 (51·4%)	62 (39·0%)	113 (46·5%)	444 (48·9%)
	≥30		80 (43·7%)	157 (48·6%)	97 (61·0%)	130 (53·5%)	464 (51·1%)
	Missing data		43	55	26	45	169
Smoking status							
	Never smoker		111 (59·4%)	166 (54·2%)	80 (53·3%)	148 (59·7%)	505 (56·7%)
	Ex-smoker		69 (36·9%)	136 (44·4%)	68 (45·3%)	97 (39·1%)	370 (41·5%)
	Current smoker		7 (3·7%)	4 (1·3%)	2 (1·3%)	3 (1·2%)	16 (1·8%)
	Missing data		39	72	35	40	186
Comorbidities							
	Number		1 (0–3)	2 (0–3)	1 (0–3)	1 (0–3)	2 (0–3)
		0	77 (34·1%)	97 (25·7%)	49 (26·5%)	92 (31·9%)	315 (29·2%)
		1	45 (19·9%)	75 (19·8%)	44 (23·8%)	58 (20·1%)	222 (20·6%)
		≥2	104 (46·0%)	206 (54·5%)	92 (49·7%)	138 (47·9%)	540 (50·1%)
	Cardiovascular		74 (32·7%)	176 (46·6%)	82 (44·3%)	123 (42·7%)	455 (42·2%)
	Respiratory		56 (24·8%)	105 (27·8%)	54 (29·2%)	69 (24·0%)	284 (26·4%)
	Type 2 diabetes*		30 (13·3%)	80 (21·2%)	40 (21·6%)	63 (21·9%)	213 (19·8%)
	Neuro-psychiatric		40 (17·7%)	60 (15·9%)	37 (20·0%)	44 (15·3%)	181 (16·8%)
	Renal and endocrine		23 (10·2%)	48 (12·7%)	11 (5·9%)	31 (10·8%)	113 (10·5%)
**Admission**							
Duration, days			2 (1–6)	6 (4–9)	10 (6–15)	33 (21–53)	9 (4–21)
	Missing data		0	0	0	1	1
PCR-positive for COVID-19			176 (84·2%)	319 (90·6%)	156 (92·3%)	243 (90·3%)	894 (89·5%)
	Missing data		17	26	16	19	78
Systemic steroids			22 (10·2%)	115 (31·5%)	57 (32·8%)	107 (45·9%)	301 (30·5%)
	Missing data		11	13	11	55	90
Antibiotic therapy			103 (47·5%)	313 (85·1%)	165 (91·7%)	260 (96·7%)	841 (81·3%)
	Missing data		9	10	5	19	43
Anticoagulation†			32 (15·1%)	89 (24·5%)	63 (35·4%)	137 (56·8%)	321 (32·3%)
	Missing data		14	14	7	47	82

Data are n (%), median (IQR), or mean (SD). Percentages are calculated by category after exclusion of missing data for that variable. WHO classes are as follows: 3–4=no continuous supplemental oxygen needed; 5=continuous supplemental oxygen only; 6=continuous or bi-level positive airway pressure ventilation or high-flow nasal oxygen; and 7–9=invasive mechanical ventilation or other organ support. IMD=Index of Multiple Deprivation. BMI=body-mass index.

* Eight participants with type 1 diabetes (of which six were in WHO class 7–9) are included within the renal and endocrine comorbidity category.

† Therapeutic dose anticoagulation; does not include intermediate doses that were not recorded.

The primary outcome measures were assessed at a median of 5·9 months (IQR 4·9–6·5) after discharge from hospital (table 2). More than 50% of the cohort were obese (appendix 1 pp 18–20). Only 239 (28·8%) of 830 individuals with available data described themselves as fully recovered. Of those working before COVID-19, 113 (17·8%) of 641 were no longer working, and 124 (19·3%) of 641 experienced a health-related change in their occupational status. Employment change was most associated with WHO class 7–9; 54 (47·8%) of 113 were no longer working and 68 (54·8%) of 124 experienced a health-related change in occupational status (appendix 1 p 16). 158 (19·6%) of 806 patients reported a new disability assessed by the WG-SS.

**Table 2:** Primary outcome measures including patient-reported outcome measures and physiological and biochemical tests, stratified by acute illness severity

		**WHO class 3–4 (n=226)**	**WHO class 5 (n=378)**	**WHO class 6 (n=185)**	**WHO class 7–9 (n=288)**	**Total (n=1077)**	**Available data, n (%)**
Time to review from discharge, days		182·5 (159·0–200·0)	168·0 (136·2–192·0)	176·0 (139·0–191·0)	179·0 (156·8–198·0)	176·0 (147·0–196·0)	1077 (100·0%)
Recovered from COVID-19?*							
	Yes	51 (30·9%)	102 (36·3%)	41 (28·5%)	45 (18·8%)	239 (28·8%)	830 (77·1%)
	No	75 (45·5%)	126 (44·8%)	65 (45·1%)	163 (67·9%)	429 (51·7%)	830 (77·1%)
	Not sure	39 (23·6%)	53 (18·9%)	38 (26·4%)	32 (13·3%)	162 (19·5%)	830 (77·1%)
	Missing data	61	97	41	48	247	**..**
Symptoms							
	Symptom count	10·0 (4·0–19·0)	7·0 (3·0–13·0)	8·0 (4·0–16·0)	9·0 (5·0–16·0)	9·0 (4·0–16·0)	861 (79·9%)
	GAD7 >8	57 (26·8%)	72 (19·9%)	44 (25·3%)	80 (28·4%)	253 (24·5%)	1031 (95·7%)
	PHQ-9 ≥10†	64 (30·2%)	79 (21·9%)	49 (28·0%)	90 (32·0%)	282 (27·4%)	1029 (95·5%)
	PCL-5 ≥38†	29 (13·6%)	31 (8·5%)	21 (12·0%)	45 (16·3%)	126 (12·2%)	1030 (95·6%)
	Dyspnoea-12	7·2 (9·4)	5·5 (7·7)	6·5 (8·8)	6·5 (8·8)	6·3 (8·6)	1017 (94·4%)
	FACIT fatigue*	18·5 (14·3)	14·6 (12·1)	16·4 (13·1)	18·5 (13·4)	16·8 (13·2)	1036 (96·2%)
Cognitive impairment							
	MoCA <23	25 (13·5%)	66 (21·0%)	19 (12·8%)	40 (16·7%)	150 (16·9%)	888 (82·5%)
Physical performance							
	SPPB ≤10	93 (46·7%)	153 (44·9%)	68 (40·5%)	134 (51·1%)	448 (46·2%)	970 (90·1%)
	ISWT % predicted†	50·4 (37·8)	50·1 (38·7)	44·7 (32·4)	39·4 (31·4)	46·2 (35·8)	634 (58·9%)
Organ function							
	FEV_1_ <80% predicted†	26 (28·6%)	43 (26·1%)	23 (28·4%)	58 (39·5%)	150 (31·0%)	484 (44·9%)
	FVC <80% predicted†	30 (33·0%)	43 (26·4%)	25 (30·9%)	62 (42·5%)	160 (33·3%)	481 (44·7%)
	TLCO <80% predicted*	3 (15·8%)	19 (30·2%)	6 (19·4%)	30 (53·6%)	58 (34·3%)	169 (15·7%)
	KCO <80% predicted	2 (10·5%)	7 (10·9%)	2 (6·2%)	5 (8·5%)	16 (9·2%)	174 (16·2%)
	BNP ≥100 ng/L or NT-BNP ≥400 ng/L	8 (5·8%)	15 (7·2%)	8 (8·0%)	15 (8·5%)	46 (7·4%)	621 (57·7%)
	HbA_1C_ ≥6·0% (DCCT/NGSP)‡	37 (27·2%)	90 (42·3%)	39 (41·1%)	47 (28·1%)	213 (34·9%)	611 (56·7%)
Systemic inflammation							
	CRP >5 mg/L	36 (21·3%)	59 (20·6%)	26 (20·0%)	59 (27·1%)	180 (22·4%)	804 (74·7%)

Data are n (%), median (IQR), or mean (SD). Percentages are calculated by category after exclusion of missing data for that variable. p values show the effect of illness severity on outcome measure. Patient outcomes were assessed at a median of 5·9 months (IQR 4·9–6·5) after hospital discharge. WHO classes are as follows: 3–4=no continuous supplemental oxygen needed; 5=continuous supplemental oxygen only; 6=continuous or bi-level positive airway pressure ventilation or high-flow nasal oxygen; and 7–9=invasive mechanical ventilation or other organ support. GAD7=Generalized Anxiety Disorder 7-item scale. PHQ-9=Patient Health Questionnaire-9. PCL-5=Post Traumatic Stress Disorder Checklist. FACIT fatigue=Functional Assessment of Chronic Illness Therapy Fatigue Scale. SPPB=short physical performance battery. ISWT=incremental shuttle walk test. CFS=Clinical Frailty Scale. MoCA=Montreal Cognitive Assessment. FVC=forced vital capacity. TLCO=transfer capacity of the lung for carbon monoxide. KCO=carbon monoxide transfer coefficient. BNP=brain natriuretic peptide. NT-BNP=N-terminal BNP. HbA_1C_=glycated haemoglobin. DCCT/NGSP=Diabetes Control and Complications Trial/National Glycohemoglobin Standardization Program. eGFR=estimated glomerular filtration rate. CRP=C-reactive protein.

* p<0·0001.

† p<0·05.

‡ p<0·01.

**Figure 2:**
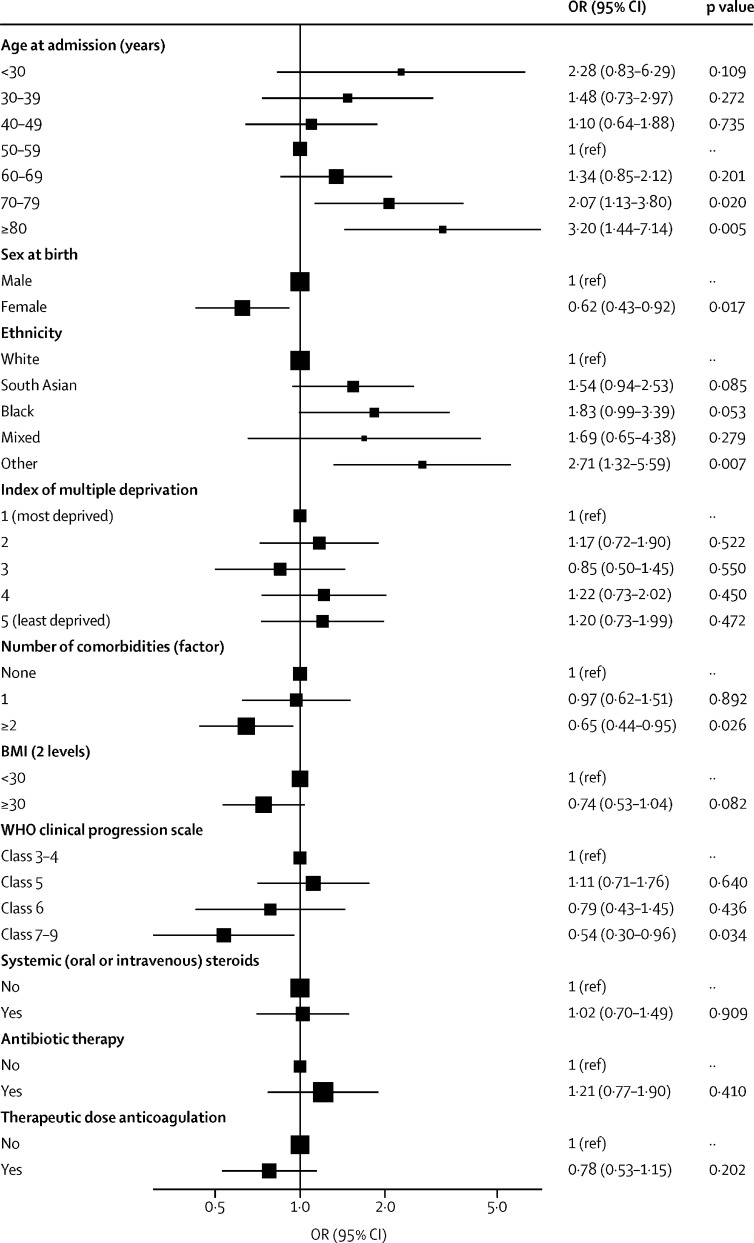
Forest plot of the patient and admission characteristics associated with patient-perceived recovery after hospitalisation for COVID-19 ORs were calculated using hierarchical multivariable logistic Regression, with admission hospital incorporated as a random effect, and multiple imputation. Patient-perceived recovery from COVID-19 was assessed at a median of 5·9 months (IQR 4·9–6·5) after discharge from hospital. Patient recovery was assessed by asking the question “Do you feel fully recovered?” and patients could respond “Yes”, “No”, or “Unsure”. ORs are presented on a log scale; bars represent 95% CIs. BMI=body-mass index. OR=odds ratio.

Factors associated with worse recovery, defined by patient-perceived recovery, were female sex, the presence of two or more pre-existing co-morbidities, and WHO class 7–9 during the acute illness (figure 2). Age had a non-linear association, with age groups <30 years and >70 years perceiving better recovery than those aged 50–59 years (figure 2). Recovery was associated with a BMI of less than 30 kg/m^2^ in the univariable and multivariable analyses, but not in imputed models (appendix 1 pp 21–22). There was no association between receiving systemic steroids during admission and recovery, nor between discharge to review time and recovery (appendix 1 p 33). The findings between the imputed and non-imputed models were similar (appendix 1 pp 21–22).

632 (92·8%) of 855 participants had at least one persistent symptom, with a median number of 9 symptoms (IQR 4–16; appendix 1 pp 23, 34). The ten most commonly reported persistent symptoms recorded at follow-up were aching of muscles (pain), fatigue, physical slowing down, impaired sleep quality, joint pain or swelling, limb weakness, breathlessness, pain, short-term memory loss, and slowing down in thinking (appendix pp 23–24). The number of persistent symptoms was highest in those with pre-existing comorbidities (median number of symptoms: 10 [5–17]), but was also high in those without pre-existing co-morbidity (median 7 [2–13]; appendix 1 pp 23–25).

Patient-reported outcome measures and measures of physical performance, pulmonary physiology, and biochemistry are shown in table 2, stratified by WHO Clinical Progression Scale (further details are provided in appendix 1, pp 18–20). More than 25% of the cohort had clinically significant symptoms of anxiety and depression, and 126 (12·2%) participants had symptoms of post-traumatic stress disorder. Physical performance measured as percentage of predicted ISWT was 46·2%, and 448 (46·2%) scored 10 or less on the SPPB, a marker of functional impairment. The severity of acute illness and patient-reported outcomes of mental health, breathlessness, fatigue, or pain, or cognitive impairment were mostly unrelated (table 2; appendix p 13). In WHO class 7–9, the percentage of predicted ISWT distance was lower, and there was a higher proportion of individuals with a percentage of predicted transfer capacity of the lung for carbon monoxide of less than 80%, but otherwise there were no clear association between measures of organ function at 5 months and the spectrum of acute severity of illness.

Patients reported their EQ-VAS score for overall health as being worse by 9·8 units (SD 18·9) at follow-up than before hospital admission, with the greatest decrement in WHO class 7–9 (p=0·0008). The greatest decreases for the EQ-5D-5L utility index and all the domains of the EQ-5D were also seen in WHO class 7–9 (appendix 1 pp 26–27, 35). A comparison between patient-estimated EQ-VAS pre-hospitalisation and age-adjusted population norms is shown in appendix 1 (p 36). At least a fifth of the cohort reached the threshold for a new disability, with at least one domain coded as “a lot of difficulty” or “cannot do it at all” on the WG-SS (appendix 1 p 28). 429 (56·9%) of 767 reported significantly worse symptoms of fatigue, 369 (48·1%) of 767 reported worse breathlessness, 318 (41·8%) of 761 reported worse sleep, and 291 (38·7%) of 751 reported worse pain (appendix 1 p 29).

**Figure 3:**
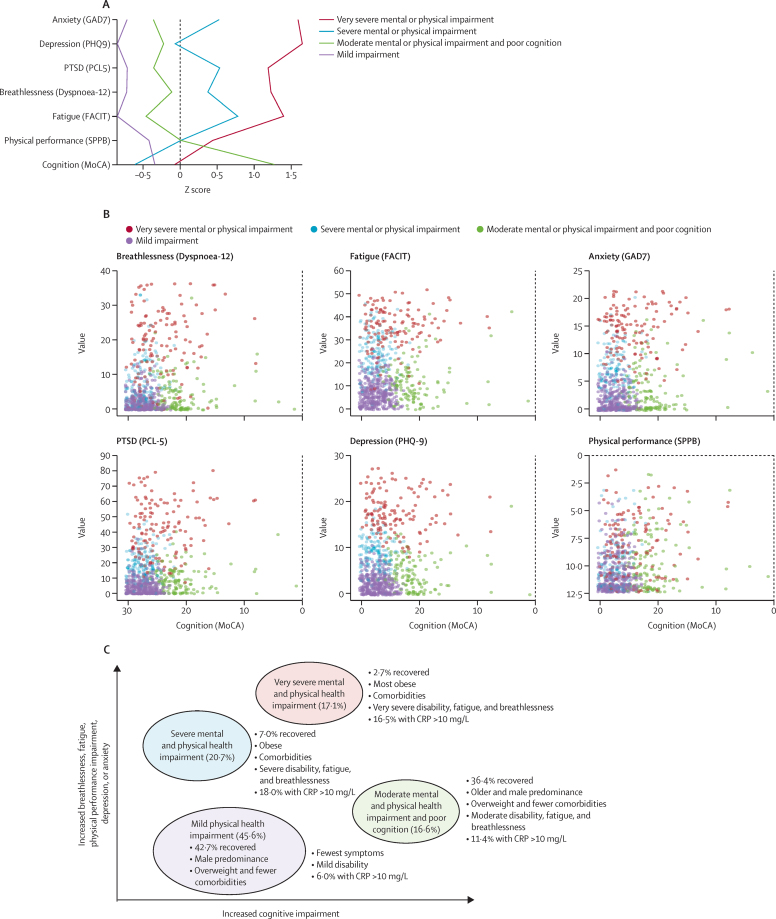
Clusters of mental, cognitive, and physical health impairments Figure shows four cluster phenotypes by Z scores, where a higher Z score indicates a higher deficit (A); clusters for cognitive impairment versus symptoms and physical function (B); and an illustration of the four cluster phenotypes with associated demographics, symptoms, and physical function (C). CRP=C-reactive protein. FACIT= Functional Assessment of Chronic Illness Therapy. GAD7=Generalized Anxiety Disorder 7-item scale. MoCA=Montreal Cognitive Assessment. PCL-5=Post Traumatic Stress Disorder Checklist. PHQ-9=Patient Health Questionnaire-9. PTSD=post-traumatic stress disorder. SPPB=short physical performance battery.

In a post-hoc analysis, we investigated phenotypes of patient-perceived recovery using patient-reported outcome measures for symptoms, mental health including post-traumatic stress disorder, MoCA for cognitive impairment, and SPPB for physical performance, and identified four clusters: very severe mental and physical impairment, severe mental and physical impairment, moderate mental and physical impairment with cognitive impairment, and mild (appendix 1 pp 30, 37–38). Of the outcomes used in the cluster analysis, all were closely associated except for cognitive impairment (figure 3, appendix pp 37–38). A comparison of demographics between clusters is shown in table 3. Respiratory and neuropsychiatric comorbidities were more common in the very severe and severe clusters, and rheumatological comorbidities were more common in the very severe cluster than in the other clusters. The Indices of Multiple Deprivation were worse in patients in the very severe and moderate clusters than in the severe and mild clusters. There was no association between cluster and WHO Clinical Progression Scale during the acute admission.

**Table 3:** Baseline assessments including patient and admission characteristics of the four recovery clusters

		**Cluster 1: very severe**	**Cluster 2: severe**	**Cluster 3: moderate and cognitive**	**Cluster 4: mild**	**Total**
Participants		131 (17·1%)	159 (20·7%)	127 (16·6%)	350 (45·6%)	767 (100·0%)
Age, years*		55·0 (10·3)	55·0 (11·2)	63·2 (13·2)	57·0 (13·2)	57·2 (12·6)
Missing data		7	3	4	2	16
Sex*						
	Female	60 (45·8%)	73 (45·9%)	46 (36·2%)	99 (28·3%)	278 (36·2%)
	Male	71 (54·2%)	86 (54·1%)	81 (63·8)	251 (71·7%)	489 (68·8%)
Ethnicity						
	White	94 (74·6%)	118 (78·1%)	77 (61·1%)	242 (71·2%)	531 (71·5%)
	South Asian	12 (9·5%)	20 (13·2%)	25 (19·8%)	60 (17·6%)	117 (15·7%)
	Black	11 (8·7%)	6 (4·0%)	15 (11·9%)	17 (5·0%)	49 (6·6%)
	Mixed	2 (1·6%)	2 (1·3%)	3 (2·4%)	6 (1·8%)	13 (1·7%)
	Other	7 (5·6%)	5 (3·3%)	5 (4·0%)	8 (2·4%)	25 (3·4%)
	Missing data	5	8	1	10	24
IMD*						
	1 (most deprived)	37 (29·4%)	24 (15·2%)	31 (25·0%)	53 (15·2%)	145 (19·2%)
	2	32 (25·4%)	36 (22·8%)	39 (31·5%)	67 (19·3%)	174 (23·0%)
	3	19 (15·1%)	29 (18·4%)	19 (15·3%)	80 (23·0%)	147 (19·4%)
	4	20 (15·9%)	32 (20·3%)	22 (17·7%)	72 (20·7%)	146 (19·3%)
	5 (least deprived)	18 (14·3%)	37 (23·4%)	13 (10·5%)	76 (21·8%)	144 (19·0%)
	Missing data	5	1	3	2	11
BMI ≥30 kg/m^2^*		79 (68·1%)	86 (60·6%)	42 (38·2%)	136 (47·2%)	343 (52·3%)
Missing data		15	17	17	62	111
Smoking status						
	Never smoker†	61 (53·0%)	70 (51·5%)	65 (60·2%)	181 (62·2%)	377 (58·0%)
	Ex-smoker	50 (43·5%)	61 (44·9%)	43 (39·8%)	109 (37·5%)	263 (40·5%)
	Current smoker	4 (3·5%)	5 (3·7%)	0 (0·0%)	1 (0·3%)	10 (1·5%)
	Missing data	16	23	19	59	117
Comorbidities						
	Number*	2 (1–4)	2 (1–3)	1 (0–3)	1 (0–2)	1 (0–3)
	Cardiovascular	65 (49·6%)	68 (42·8%)	51 (40·2%)	128 (36·6%)	312 (40·7%)
	Neuro-psychiatric*	53 (40·5%)	37 (23·3%)	12 (9·4%)	23 (6·6%)	125 (16·3%)
	Respiratory*	56 (42·7%)	47 (29·6%)	23 (18·1%)	80 (22·9%)	206 (26·9%)
	Rheumatology‡	25 (19·1%)	16 (10·1%)	15 (11·8%)	27 (7·7%)	83 (10·8%)
	Type 2 diabetes	26 (19·8%)	32 (20·1%)	24 (18·9%)	57 (16·3%)	139 (18·1%)
Hospital stay, days		12·0 (4·5–27·0)	7·0 (3·0–25·5)	10·0 (5·0–17·0)	8·0 (4·0–18·0)	9·0 (4·0–20·0)
	Missing data	0	0	0	1	1
WHO Clinical Progression Scale						
	Class 3–4	27 (20·6%)	38 (23·9%)	22 (17·3%)	67 (19·1%)	154 (20·1%)
	Class 5	33 (25·2%)	53 (33·3%)	56 (44·1%)	132 (37·7%)	274 (35·7%)
	Class 6	21 (16·0%)	24 (15·1%)	20 (15·7%)	61 (17·4%)	126 (16·4%)
	Class 7–9	50 (38·2%)	44 (27·7%)	29 (22·8%)	90 (25·7%)	213 (27·8%)
PCR-positive for COVID-19		108 (89·3%)	127 (85·8%)	107 (89·2%)	292 (90·1%)	634 (88·9%)
	Missing data	10	11	7	26	54

Data are n (%), median (IQR), or mean (SD). There was no relationship between other categories of comorbidities and cluster classification. p values represent the differences in characteristics between clusters. WHO classes are as follows: 3–4=no continuous supplemental oxygen needed; 5=continuous supplemental oxygen only; 6=continuous or bi-level positive airway pressure ventilation or high-flow nasal oxygen; and 7–9=invasive mechanical ventilation or other organ support. IMD=Index of Multiple Deprivation.

* p<0·0001.

† p<0·05.

‡ p<0·01.

The patient-reported outcomes and physical performance were different between the clusters (table 4). Impairments in lung physiology and biochemical tests used in the assessment of heart failure, renal failure, diabetes, and pre-diabetes, were observed in all the clusters, but none were discriminatory across clusters. In the whole cohort, 180 (22·4%) of 804 had persistent systemic inflammation, measured by C-reactive protein of more than 5 mg/L, which was weakly correlated with BMI (*r*=0·247; p<0·0001). Elevated C-reactive protein was more prevalent in the very severe and severe clusters, compared with the moderate and mild clusters.

**Table 4:** Primary outcome measures stratified by four recovery clusters

	**Cluster 1: very severe (n=131)**	**Cluster 2: severe (n=159)**	**Cluster 3: moderate and cognitive (n=127)**	**Cluster 4: mild (n=350)**	**Total (n=767)**	**Available data, n (%)**
Time to review from discharge, days	174·0 (136·0–198·5)	175·0 (148·0–192·0)	176·0 (152·0–192·0)	176·0 (148·2–194·0)	175·0 (147·5–194·0)	767 (100·0%)
Persistent symptoms*	114 (100·0%)	131 (98·5%)	97 (91·5%)	230 (83·6%)	572 (91·1%)	628 (81·9%)
Symptom count*	20 (16–25)	13 (8–17)	7 (3–12)	5 (2–8)	8 (4–16)	628 (81·9%)
GAD-7 >8*	118 (90·1%)	44 (27·7%)	16 (12·6%)	4 (1·1%)	182 (23·7%)	767 (100·0%)
PHQ-9 ≥10*	128 (97·7%)	60 (37·7%)	9 (7·1%)	1 (0·3%)	198 (25·8%)	767 (100·0%)
PCL-5 ≥38*	79 (60·3%)	9 (5·7%)	2 (1·6%)	0	90 (11·7%)	767 (100·0%)
Dyspnoea-12*	18·2 (9·9)	6·7 (6·4)	4·2 (5·3)	1·6 (2·5)	5·9 (8·2%)	767 (100·0%)
FACIT fatigue*	34·9 (8·9)	23·8 (8·6)	11·2 (8·4)	7·0 (5·2)	15·9 (12·9)	767 (100·0%)
BPI severity*	21·5 (8·4)	13·9 (8·6)	11·7 (10·1)	7·3 (7·6)	12·1 (9·9)	587 (76·5%)
BPI interference*	39·9 (17·1)	21·2 (14·9)	14·1 (15·8)	6·7 (9·7)	17·5 (18·3)	574 (74·8%)
SPPB (mobility disability)*	94 (71·8)	72 (45·3)	67 (52·8)	101 (28·9)	334 (43·5)	767 (100·0%)
ISWT distance, m*	278 (190)	432 (258)	415 (226)	536 (262)	452 (261)	463 (60·4%)
CFS ≥5*	26 (22·8%)	2 (1·3%)	4 (3·5%)	4 (1·2%)	36 (5·1%)	702 (91·5%)
MoCA <23*	40 (30·5%)	2 (1·3%)	82 (64·6%)	0	124 (16·2%)	767 (100·0%)
Adjusted MoCA <23*	36 (27·5%)	1 (0·6%)	70 (55·1%)	0	107 (14·0%)	767 (100·0%)
FEV_1_ % predicted†	79·8 (20·9)	91·2 (35·5)	89·5 (20·8)	91·6 (17·5)	89·3 (24·2)	359 (46·8%)
FEV_1_ <80% predicted‡	27 (49·1%)	23 (27·7%)	18 (26·1%)	34 (22·4%)	102 (28·4%)	359 (46·8%)
FVC <80% predicted‡	30 (55·6%)	25 (30·1%)	20 (29·0%)	40 (26·3%)	115 (32·1%)	358 (46·7%)
FEV_1_/FVC <0·7	4 (6·1%)	11 (11·3%)	8 (10·8%)	14 (7·6%)	37 (8·8%)	421 (54·9%)
TLCO <80% predicted	8 (40·0%)	12 (36·4%)	7 (30·4%)	18 (27·7%)	45 (31·9%)	141 (18·4%)
KCO <80% predicted†	2 (9·5%)	7 (21·2%)	3 (12·5%)	2 (3·0%)	14 (9·7%)	144 (18·8%)
BNP/NT-BNP % greater than threshold§	5 (7·2%)	4 (4·0%)	5 (6·7%)	13 (6·7%)	27 (6·2%)	439 (57·2%)
HbA_1C_ ≥6·0% (DCCT/NGSP)	27 (41·5%)	26 (27·4%)	29 (38·2%)	65 (33·0%)	147 (33·9%)	433 (56·5%)
eGFR <60 mL/min per 1·73 m^2^	16 (15·5%)	11 (8·0%)	14 (13·7%)	35 (12·9%)	76 (12·4%)	615 (80·2%)
D-dimer ≥500 ng/mL	8 (9·5%)	14 (11·4%)	11 (11·6%)	30 (12·3%)	63 (11·5%)	546 (71·2%)
D-dimer, mg/L	276·0 (348·9)	240·1 (200·9)	274·1 (269·2)	290·2 (346·5)	273·9 (306·6)	546 (71·2)
CRP >5 mg/L‡	29 (29·3%)	36 (27·9%)	17 (17·2%)	36 (14·3%)	118 (20·4%)	578 (75·4%)

Data are n (%), median (IQR), or mean (SD). Patient outcomes were assessed at a median of 5·9 months (IQR 4·9–6·5) after hospital discharge. p values represent differences between clusters. GAD7=Generalized Anxiety Disorder 7-item scale. PHQ-9=Patient Health Questionnaire-9. PCL-5=Post Traumatic Stress Disorder Checklist. FACIT fatigue=Functional Assessment of Chronic Illness Therapy Fatigue Scale. BPI=Brief Pain Inventory. SPPB=short physical performance battery. ISWT=incremental shuttle walk test. CFS=Clinical Frailty Scale. MoCA=Montreal Cognitive Assessment. Adjusted MoCA=MoCA adjusted for education. FVC=forced vital capacity. TLCO=transfer capacity of the lung for carbon monoxide. KCO=carbon monoxide transfer coefficient. BNP=brain natriuretic peptide. NT-BNP=N-terminal BNP. HbA_1C_=glycated haemoglobin. DCCT/NGSP=Diabetes Control and Complications Trial/National Glycohemoglobin Standardization Program. eGFR=estimated glomerular filtration rate. CRP=C-reactive protein.

* p<0·0001.

† p<0·05.

‡ p<0·01.

§ Threshold BNP ≥100 ng/L or NT-BNP ≥400 ng/L.

The patient-perceived recovery was lowest in the very severe (three [2·7%] of 113 patients) and severe (nine [7·0%] of 129) clusters compared with the moderate (36 [36·4%] of 99) and mild (114 [42·7%] of 267) clusters (p<0·0001; appendix 1 pp 31–32). The EQ-VAS and utility index was lowest in the very severe cluster before COVID-19, with the greatest decreases between before COVID-19 and follow-up in the very severe and severe clusters (p<0·0001). There were higher rates of new disability (assessed by WG-SS) in the very severe cluster (57 [51·8%] of 110) compared with the severe cluster (25 [20·0%] of 125), the moderate cluster (11 [11·5%] of 96), and the mild cluster (12 [4·6%] of 262; p<0·0001). The very severe cluster had a greater proportion of people no longer working after hospitalisation with COVID-19 (50·0% versus 10·0% to 16·1% across the other clusters; p<0·0001), and the greater proportion of people who experienced a change in occupation due to health reasons after COVID-19 (60·0% versus 8·7% to 19·4% across the other clusters; p<0·0001; appendix 1 p 17).

## Discussion

This is the largest study, across multiple UK centres, to comprehensively report on prospectively assessed outcomes to describe the holistic impact of COVID-19 on the medium-term health of survivors. The majority of patients had not fully recovered, had persistent symptoms, and 20% had a new disability. In the two-thirds who were working before admission to hospital, 19% had changed working status, predominately due to ill health. Not fully recovering was associated with female sex, middle age (40–59 years), two or more comorbidities, and having received mechanical ventilation. Treatment with systemic corticosteroids was not associated with recovery. The magnitude of the ongoing mental and physical health burden was substantial but, perhaps surprisingly, several aspects of the ongoing health burden were unrelated to acute severity. Measures of pulmonary transfer factor, walking performance, and changes in health-related quality of life appeared worse in those who had received mechanical ventilation, but the impact on health status by symptoms (breathlessness, fatigue, pain, anxiety, and depression) and other measures of organ function were unrelated to acute illness severity. This finding led us to define further possible recovery phenotypes using cluster analysis, including validated tools for breathlessness, fatigue, mental health, cognitive impairment, and physical function. We identified four recovery clusters that tracked with severity of both mental and physical health impairment, except for cognitive impairment, which was largely independent. Whether these clusters have different underlying mechanisms and warrant different treatments and clinical pathways needs to be determined.

Our finding of 20–30% recovery at 6 months after hospitalisation for COVID-19 is consistent with previous reports.^4^, ^22^ By contrast, in adults who were not hospitalised, recovery after COVID-19 is approximately 70–90% in most studies at 3 months,^23^ suggesting that severity of the acute illness warranting hospitalisation is associated with a lower likelihood of recovery. The persistent symptom burden and proportion of patients not recovering at 6 months after hospitalisation from COVID-19 appears greater than was previously reported for older adults (mean age 62 years) hospitalised with community acquired pneumonia, who had a median recovery time of 3 weeks.^24^

We report an inconsistent relationship between the severity of the different health impairments and the severity of the acute illness, implying different underlying mechanisms. The degree of acute lung injury largely determines the level of respiratory support during the acute illness; it is therefore unsurprising that measures of pulmonary and physical function at 6 months are associated with the severity of acute illness. However, the same relationship is not observed for markers of cardiac or renal impairment, nor for the impact of persistent symptoms. We therefore suggest that although there is evidence of a post-intensive care unit syndrome consistent with other illnesses, other mechanisms are causing the ongoing symptoms across the acute severity spectrum, supported by the magnitude of persistent symptoms seen in those who were never hospitalised.^25^ Based on our data, holistic post-hospital care pathways should be proactive, available to all hospitalised patients and not solely those who received ventilatory support, and should include symptom assessment.

Some of our reported associations with reduced patient-perceived recovery contrast with those associated with worse outcome during the acute illness. Consistent with a previous report for community-managed COVID-19,^23^ we found female sex was associated with not recovering, in contrast to male sex being a risk factor for more severe acute illness. Autoimmunity is more common in women older than 40 years, and both anti-cytokine and tissue-specific autoantibodies have been implicated in post-COVID syndromes;^26^, ^27^, ^28^ this is one possible explanation for the association that needs further investigation. Despite greater morbidity and mortality greater acute COVID-19 infection and increased risk post-discharge of cardiometabolic and respiratory events in ethnic minorities,^29^, ^30^, ^31^ there was no association with absence of full recovery. Age had a non-linear relationship with recovery, with those who were younger or older having a higher likelihood of recovery, whereas poor recovery was associated with middle age (40–59 years). Having two or more comorbidities was associated with both increased risk of severe acute illness and subsequent poor recovery post hospitalisation, and similarly, obesity appears to be a risk factor for both. Systemic corticosteroid therapy for the acute illness reduces mortality in those with more severe acute disease,^32^ but was not associated with post-discharge medium-term recovery.

The four recovery clusters we identified largely corresponded to severity of mental and physical health impairment, but cognitive impairment was largely independent. The independence of cognitive impairment has also been noted in the recovery trajectory for survivors of adult respiratory distress syndrome.^33^, ^34^ Of note, the clusters were not closely related to acute illness severity in our study, further supporting the view that severity of persistent physical and mental ill health and cognitive impairment are due to mechanisms other than those directly related to severity of the acute lung injury (assessed in our cohort by the highest level of respiratory support using the WHO Clinical Progression Scale). Pre-COVID-19 poor health status and comorbidities including obesity were particularly a feature of the very severe cluster and, to a lesser extent, the severe cluster, compared with the moderate and mild clusters. However, changes in EQ-VAS and utility index showed that the greatest impact on health status was in the very severe and severe clusters, even accounting for poorer health status before COVID-19. Objective tests of organ function were not discriminatory across the clusters except for percentage of predicted FEV_1_ and C-reactive protein levels. The percentage of predicted FEV_1_ was lower in the very severe cluster than in other clusters without evidence of airflow obstruction, suggesting that airflow restriction was possibly due to lung fibrosis or in part due to extra-thoracic restriction secondary to obesity.

C-reactive protein was greater than 5 mg/L at 5 months in more than 22% of the cohort and was particularly elevated in the very severe and severe clusters compared with the mild cluster, possibly due to persistent systemic inflammation. We found only a weak correlation between BMI and C-reactive protein level, suggesting that although the elevated C-reactive protein in the very severe, severe, and moderate clusters might be partly due to increased BMI, this is unlikely to fully explain the increased systemic inflammation. Therefore, the magnitude of the physical and mental health impact, the heterogeneity of the cognitive impairment between and within these clusters, and the impact of persistent inflammation and its effect on the immunological response require more understanding of possible underlying mechanisms. In terms of clinical care, these four cluster phenotypes point to the potential of stratifying follow-up care and interventions according to the severity and causes of the impairments. For example, those in the mild cluster might need less intense follow-up than those in the very severe cluster, and those with persistent inflammation or obesity might benefit from anti-inflammatory therapy or strategies to promote weight loss.^35^

Beyond the impact on health, working status changed for one in five patients, and a similar proportion experienced a health-related change in occupation. This impact on occupation was most marked in the group that had required mechanical ventilation, similar to findings from intensive therapy unit survivorship studies.^36^ In the recovery clusters, impact on occupation was most striking in the very severe cluster, from more than 60% working before COVID-19 to around 50% having changed occupation after hospitalisation, almost entirely due to poor health.^36^ This societal impact is clearly substantial in those hospitalised but is also highly relevant for non-hospitalised cases of COVID-19.

The strengths of our large, multicentre study include the most comprehensive assessment of in-clinic and patient-reported outcomes to date, highlighting the inconsistent relationship between the severity of ongoing health impairments and the severity of the acute illness between different health domains, and enabling symptom cluster phenotyping. However, this study has a number of limitations. The patients represent a small sample of the total number of patients discharged from hospital after having COVID-19 in the UK. The study population is younger than the overall population in the UK that survived hospitalisation for COVID-19,^37^ and only included those able to attend clinic visits and undertake the study procedures. This acquisition bias might under-represent the most severely affected patients, but conversely, those patients with ongoing symptoms might have been more willing to participate. Missing data were variable across the outcomes, with potential selection bias. The variation in time from discharge to assessment was broad and, although it did not affect patient-perceived recovery, the stability of the cluster phenotypes over time needs to be understood. The results need validating in a separate cohort, alongside understanding the association between cluster membership and future health outcomes. The patient-reported outcomes and physical and biological tests assessed cross-sectionally do not allow for a clear differentiation between the contribution from premorbid disease versus emergent impaired health status and symptoms. The finding of no association between corticosteroids prescribed for the acute illness and recovery might be confounded by a temporal change in practice patterns and in-hospital mortality during the study enrolment period. We did not account for multiple testing, which means that some statistically significant findings could be false positives; p values are provided in full for the reader to interpret (appendix pp 14–32). Validation in an independent cohort is required to confirm associations.

The outcome measures for this analysis had some limitations. For example, we did not have access to highly sensitive C-reactive protein, potentially underestimating the proportion of patients with elevated C-reactive protein. Although the MoCA is a validated, global measure of cognitive impairment, more in-depth testing is required to understand the precise deficits people are experiencing when describing “brain fog”. There may be other ongoing sequelae not captured by the outcome measures used. We used patient-perceived health status before hospital admission as a surrogate baseline for the symptom visual analogue scales, the EQ-5D-5L, and the WG-SS tool; these retrospective measures could be subject to recall bias.^38^ Our patient-centred definition for recovery in this report is a subjective definition based on patient perception and is likely to reflect who might seek post-COVID care. Although patient-perceived recovery might be affected by recall bias of pre-hospitalisation health, a similar five-point Likert scale has subsequently been developed and recommended as a core outcome measure for COVID-19 studies.^39^ In addition, patient-perceived recovery corresponded well to the overall burden of disease identified in the recovery phenotypes, but how pre-existing comorbidities affect patient-perceived recovery is unknown. Patient-perceived recovery will fail to identify pathological changes that have not yet led to clinical expression, but which might become overt in later follow-up. Further comparisons are also required with recovery after other acute respiratory infections leading to hospitalisation and with those infected with COVID-19 but not hospitalised, to understand the specificity of our findings to COVID-19 versus other critical illnesses and between those hospitalised versus those managed in the community.

Further analysis of the trajectory of recovery and linkage to primary and secondary health records within PHOSP-COVID will enable further discrimination. Notwithstanding these limitations, the magnitude of burden of physical and mental health is substantial post hospitalisation for COVID-19.

This is the first report from the PHOSP-COVID study, which includes biosampling for further immunological, multi-omic, and imaging endpoints, including multi-modality MRI. These data will enable further analysis of systemic and organ-specific inflammation and damage. Further study of the trajectory of recovery, coupled with this greater mechanistic understanding, will inform a precision medicine approach to the clinical management of hospitalised COVID-19 survivors.

In conclusion, the majority of survivors of a hospital admission with COVID-19 are not fully recovered at 5 months and have a substantial and diverse physical and mental health burden and negative effects on employment. We identified key factors associated with recovery and four distinct recovery phenotypes using cluster analysis according to mental, cognitive, and physical health. Our findings support the need for a proactive approach to clinical follow-up, with a holistic assessment to include symptoms of mental and physical health, and validated assessment of cognitive impairment. The four severity clusters highlight the potential to stratify care and the need for wide access to COVID-19 holistic clinical services, including mental health, memory and cognition, and rehabilitation services.

## Data sharing

The protocol, consent form, definition and derivation of clinical characteristics and outcomes, training materials, regulatory documents, information about requests for data access, and other relevant study materials are available online.

## Declaration of interests

JDC reports grants and personal fees from AstraZeneca, Boehringer-Ingelheim, GlaxoSmithKline, Novartis, and Insmed, personal fees from Chiesi, Zambon, Janssen, and Grifols, and grants from Gilead Sciences, outside the submitted work. TC reports grants from Guy's and St Thomas' Charity, fees from workshops, and fees from writing self-help books on fatigue, outside the submitted work. NE received a donation of SARS-CoV-2 lateral flow antigen test kits from Mologic, in relation to an unrelated COVID-19 project. Neither NE nor his institution have received any financial compensation and he has no financial relationship of any kind with Mologic. RAE reports grants from GlaxoSmithKline during the conduct of the study; and grants from the National Institute for Health Research (NIHR) and personal fees from GlaxoSmithKline, AstraZeneca, and Chiesi, outside the submitted work. AH reports personal fees from Vertex Pharmaceuticals, Mylan Healthcare, and the Cystic Fibrosis Foundation, and grants from JP Moulton Trust and NIHR, outside the submitted work. LGH reports receiving sponsorship for attending international scientific meetings from AstraZeneca, Boehringer Ingelheim, Chiesi, GlaxoSmithKline, and Napp Pharmaceuticals, personal fees from Novartis, Hoffman la Roche/Genentech, Sanofi, Evelo Biosciences, GlaxoSmithKline, AstraZeneca, Teva, Theravance, and Circassia, and grants from Medimmune, Novartis UK, Roche/Genentech, GlaxoSmithKline, Amgen, Genentech/Hoffman la Roche, AstraZeneca, Medimmune, Aerocrine, and Vitalograph, outside the submitted work. NH reports that his research group has received unrestricted grants (managed by Guy's & St Thomas' Foundation Trust) from Philips and Resmed. Philips are contributing to the development of the MYOTRACE technology. SH reports personal fees and fees to institution for advisory boards and consultancy from Novo Nordisk, Eli Lilly, Zealand Pharma, and Sanofi Aventis, outside the submitted work. JJ reports personal fees from Boehringer Ingelheim, Roche, GlaxoSmithKline, and NHSX, outside the submitted work. RGJ reports personal fees and research funding from Biogen, personal fees from Galapagos, Heptares, Boehringer Ingelheim, Pliant, Roche/InterMune, MedImmune, PharmAkea, Bristol Myers Squibb, Chiesi, Roche/Promedior, Veracyte, and GlaxoSmithKline research funding from Galecto, collaborative award from RedX and Nordic Biosciences, and was an advisory board member for NuMedii, outside the submitted work. RGJ is supported by an NIHR Professorship (RP-2017-08-ST2-014) and is a trustee for Action for Pulmonary Fibrosis. GM reports grants from AstraZeneca, outside the submitted work. PJMO reports grants from the Medical Research Council (MRC), the EU, and NIHR, and personal fees from Pfizer, Nestle, and Janssen, outside the submitted work. PP reports a grant from NIHR, outside the submitted work. MRo reports a senior clinical fellowship as part of research training and a 1-year post working in Pharma Development Neurosciences with Roche Pharmaceuticals, outside the submitted work. ADS reports grants and personal fees from AstraZeneca, Bayer, Boehringer, Chiesi, Forest Laboratories, GlaxoSmithKline, Grifols, Insmed, MedImmune, Novartis, Pfizer, and 30T, outside the submitted work. MGS reports grants from NIHR, MRC, and the Health Protection Research Unit in Emerging & Zoonotic Infections, University of Liverpool, during the conduct of the study; and reports being a minority owner and chair of the infectious disease scientific advisory board for Integrum Scientific, outside the submitted work. AShe reports being a Member of the Scottish Government's Chief Medical Officer's COVID-19 Advisory Group. MT reports personal fees from Merck Sharp & Dohme and GlaxoSmithKline, and grants and personal fees from Bayer and Actelion, during the conduct of the study. LVW reports grants from GlaxoSmithKline and Orion, outside the submitted work. All other authors declare no competing interests.
