# Correction to *Lancet Respir Med* 2021; 9: 1275–87

**DOI:** 10.1016/S2213-2600(21)00540-3

**Published:** 2022-01

*Evans RA, McAuley H, Harrison EM, et al. Physical, cognitive, and mental health impacts of COVID-19 after hospitalisation (PHOSP-COVID): a UK multicentre, prospective cohort study.* Lancet Respir Med *2021;* 9: *1275–87*—Appendix 1 of this Article has been corrected as of Dec 1, 2021.

