# Correction to *Lancet Respir Med* 2021; 9: 1275–87

**DOI:** 10.1016/S2213-2600(24)00142-5

**Published:** 2024-06

*Evans RA, McAuley HJC, Harrison EM, et al. Physical, cognitive, and mental health impacts of COVID-19 after hospitalisation (PHOSP-COVID): a UK multicentre, prospective cohort study.* Lancet Respir Med *2021; **9:** 1275–87*—For this Article, the middle initials for coauthor Hamish J C McAuley were added and the PHOSP-COVID Collaborative Group list and appendix 1 have been updated. These corrections have been made to the online version as of May 29, 2024.

