# Supplementary material for: Physical, cognitive, and mental health impacts of COVID-19 after hospitalisation (PHOSP-COVID): a UK multicentre, prospective cohort study
Source: Lancet Respir Med. 2021 Nov;9(11):1275–87. doi: 10.1016/S2213-2600(21)00383-0 (PMC8497028; doi:10.1016/S2213-2600(21)00383-0)
Supplement: Supplementary appendix 1 [file mmc1.pdf]

THE LANCET
Respiratory Medicine

Supplementary appendix 1

This appendix formed part of the original submission and has been peer reviewed. We post it as supplied by the authors.

Evans RA, McAuley H, Harrison EM, et al. Physical, cognitive, and mental health impacts of COVID-19 after hospitalisation (PHOSP-COVID): a UK multicentre, prospective cohort study. *Lancet Respir Med* 2021; published online Oct 7. https://doi.org/10.1016/S2213-2600(21)00383-0.

Supplement

Contents

The PHOSP-COVID Collaborative Group 1

Supplementary Methods 10

Supplementary Data – Results 14

Tables 14

Figures 33

References 39

The PHOSP-COVID Collaborative Group

Writing Committee

R A Evans[1*], H McAuley[1], Prof E M Harrison[2], A Shikotra[1], A Singapuri[1], M Sereno[1], O Elneima[1], A B Docherty[2], N I Lone[3,4], O C Leavy[5], L Daines[3], J K Baillie[6,7], Prof J S Brown[8], Prof T Chalder[9], Prof A De Soyza[10,11], Prof N Diar Bakerly[12,13], N Easom[14], Prof J R Geddes[15,16], N J Greening[1], Prof N Hart[17], Prof L G Heaney[18,19], Prof S Heller[20], L Howard[21], Prof J R Hurst[22], J Jacob[23,24], Prof R G Jenkins[25], C Jolley[26], S Kerr[6], Prof O M Kon[27,25], Prof K Lewis[28,29,30], Prof J M Lord[31], Prof G P McCann[32,33], Prof S Neubauer[34,35], Prof P J M Openshaw[25], D Parekh[31,36], P Pfeffer[37,38], Prof N M Rahman[39], B Raman[34], M Richardson[1], M Rowland[40], Prof M G Semple[41,42], Prof A M Shah[43], Prof S J Singh[1], Prof A Sheikh[3], D Thomas[44], M Toshner[45,46], Prof J D Chalmers[47], Prof L P Ho[48], A Horsley[49], M Marks[50,51], K Poinasamy[52], Prof L V Wain[5,1*], Prof C E Brightling[1*]

*first and joint last authors contributed equally

The affiliations of the members of the writing committee are as follows:

[1]The Institute for Lung Health, Leicester NIHR Biomedical Research Centre, University of Leicester, Leicester, United Kingdom, [2]Centre for Medical Informatics, The Usher Institute, University of Edinburgh, Edinburgh, United Kingdom, [3]Usher Institute, University of Edinburgh, Edinburgh, United Kingdom, [4]Royal Infirmary of Edinburgh, NHS Lothian, Edinburgh, United Kingdom, [5]Department of Health Sciences, University of Leicester, Leicester, United Kingdom, [6]Roslin Institute, University of Edinburgh, Edinburgh, United Kingdom, [7]Intensive Care Unit, Royal Infirmary of Edinburgh, Edinburgh, United Kingdom, [8]UCL Respiratory, Department of Medicine, University College London, Rayne Institute, London, United Kingdom, [9]Department of Psychological Medicine, Institute of Psychiatry, Psychology and Neuroscience, King's College London, London, United Kingdom, [10]Population Health Sciences Institute, Newcastle University, Newcastle Upon Tyne, United Kingdom, [11]Newcastle upon Tyne Teaching Hospitals Trust, Newcastle upon Tyne, United Kingdom, [12]Manchester Metropolitan University, Manchester, United Kingdom, [13]Salford Royal NHS Foundation Trust, Manchester, United Kingdom, [14]Infection Research Group, Hull University Teaching Hospitals, Hull, United Kingdom, [15]NIHR Oxford Health BRC, Department of Psychiatry, University of Oxford, Warneford Hospital, Oxford, United Kingdom, [16]Oxford Health NHS Foundation Trust, Oxford, United Kingdom, [17]Lane Fox Respiratory Service, Guy's and St Thomas' NHS Foundation Trust, London, United Kingdom, [18]Wellcome-Wolfson Institute for Experimental Medicine, Queens University Belfast, United Kingdom, [19]Belfast Health & Social Care Trust, Belfast, United Kingdom, [20]Department of Oncology and Metabolism, University of Sheffield, Sheffield, United Kingdom, [21]Imperial College Healthcare NHS Trust, London, United Kingdom, [22]UCL Respiratory, Royal Free Campus, University College London, London, United Kingdom, [23]Centre for Medical Image Computing, University College London, London, United Kingdom, [24]Lungs for Living Research Centre, University College London, London, United Kingdom, [25]National Heart and Lung Institute, Imperial College London, London, United Kingdom, [26]Centre for Human and Applied Physiological Sciences, School of Basic and Medical Biosciences, Faculty of Life Sciences and Medicine, King's College London, London, United Kingdom, [27]Respiratory Medicine, Imperial College Healthcare NHS Trust, London, United Kingdom, [28]Hywel Dda University Health Board, Wales, United Kingdom, [29]University of Swansea, Wales, United Kingdom, [30]Respiratory Innovation Wales, Wales, United Kingdom, [31]Institute of Inflammation and Ageing, University of Birmingham, Birmingham, United Kingdom, [32]Department of Cardiovascular Sciences, University of Leicester, Leicester, United Kingdom, [33]Leicester NIHR Biomedical Research Centre, University of Leicester, Leicester, United Kingdom, [34]Division of Cardiovascular Medicine, Radcliffe Department of Medicine, University of Oxford, Oxford, United Kingdom, [35]NIHR Biomedical Research Centre, John Radcliffe Hospital, Oxford, United Kingdom, [36]Department of Acute Medicine, Queen Elizabeth Hospital Birmingham, Birmingham, United Kingdom, [37]Barts Health NHS Trust, London, United Kingdom, [38]Queen Mary University of London, London, United Kingdom, [39]Oxford Respiratory Trials Unit, University of Oxford, Oxford, United Kingdom, [40]Kadoorie

Centre for Critical Care Research, Nuffield Department of Clinical Neurosciences, University of Oxford, Oxford, United Kingdom, [41]NIHR Health Protection Research Unit in Emerging and Zoonotic Infections, Institute of Infection, Veterinary and Ecological Sciences, University of Liverpool, United Kingdom, [42]Respiratory Medicine, Alder Hey Children's Hospital, Liverpool, United Kingdom, [43]King's College London British Heart Foundation Centre and King's College Hospital NHS Foundation Trust, London, UK [44]Immunology and Inflammation, Imperial College, London, United Kingdom, [45]Cambridge NIHR BRC, Cambridge, United Kingdom, [46]NIHR Cambridge Clinical Research Facility, Cambridge, United Kingdom, [47]University of Dundee, Ninewells Hospital and Medical School, Dundee, United Kingdom, [48]MRC Human Immunology Unit, University of Oxford, Oxford, United Kingdom, [49]Division of Infection, Immunity & Respiratory Medicine, Faculty of Biology, Medicine and Health, University of Manchester, Manchester, United Kingdom, [50]Department of Clinical Research, London School of Hygiene & Tropical Medicine Keppel Street, London, United Kingdom, [51]Hospital for Tropical Diseases, University College London Hospital, London, United Kingdom, [52]Asthma UK and British Lung Foundation, London, United Kingdom

Steering Committee

Co-chairs D Lomas, E Sapey*, Institution representatives* C Berry, C E Bolton, N Brunskill, E R Chilvers, R Djukanovic, Y Ellis, D Forton, N French, J George, N A Hanley, N Hart, L McGarvey, N Maskell, H McShane, M Parkes, D Peckham, P Pfeffer, A Sayer, A Sheikh, A A R Thompson, N Williams and core management group representation

Executive Board

Chair C E Brightling, representation from the core management group, each working group and platforms

Core Management Group

Chief Investigator C E Brightling, *Members* R A Evans (Lead Co-I), L V Wain (Lead Co-I), J D Chalmers, L P Ho, A Horsley, M Marks, K Poinasamy, B Raman, A Singapuri, A Shikotra,

Working Groups

Airways

L G Heaney (*Co-Lead*), A De Soyza (*Co-Lead*), D Adeloye, C E Brightling, J S Brown, J Busby, J D Chalmers, L Daines, O Elneima, J Hurst, P Novotny, P Pfeffer, K Poinasamy, J Quint, I Rudan, E Sapey, A Sheikh, S Siddiqui, S Walker

Brain

M Hotopf *(Co-Lead),* J R Geddes (*Co-Lead*), K Abel, R Ahmed, L Allan, C Armour, D Baguley, D Baldwin, C Ballard, J Bambrough, K Bhui, G Breen, M Broome, T Brugha, E Bullmore, D Burn, J Cavanagh, T Chalder, D Clark, A David, B Deakin, H Dobson, B Elliott, J Evans, R Francis, E Guthrie, P Harrison, M Henderson, A

Hosseini, N Huneke, M Husain, T Jackson, I Jones, T Kabir, P Kitterick, A Korszun, I Koychev, J Kwan, A Lingford-Hughes, C Mackay, P Mansoori, H McAllister-Williams, K McIvor, B Michael, L Milligan, R Morriss, E Mukaetova-Ladinska, T Nicholson, S Paddick, C Pariante, J Pimm, K Saunders, M Sharpe, G Simons, R Upthegrove, S Wessely

Cardiac

G P McCann (*Co-Lead*), C Antoniades, R Bell, A Bularga, C Berry, K Channon, P Chowienczyk, J Greenwood, A Hingorani, A Hughes, K Khunti, C Kotanidis, J Mayet, N Mills, A J Moss, S Neubauer, D Newby, B Raman, N Samani, A Saratzis, N Sattar, A Shah, C Sudlow, M Toshner, R Touyz, B Williams, C Xie

Immunology

T Hussell (*Co-Lead*), P J M Openshaw (*Co-Lead*), D Altmann, J K Baillie, R Batterham, H Baxendale, N Bishop, C E Brightling, P C Calder, R A Evans, J L Heeney, P Klenerman, J M Lord, P Moss, S L Rowland-Jones, W Schwaeble, M G Semple, R S Thwaites, L Turtle, L V Wain, S Walmsley, D Wraith

Intensive Care

M J Rowland (*Co-Lead*), A Rostron (Co-Lead), J K Baillie, B Connolly, A B Docherty, N I Lone, D F McAuley, D Parekh, J Simpson, C Summers

Lung Fibrosis

R G Jenkins (*Co-Lead*), J Porter (*Co-Lead*), R Allen, R Aul, J K Baillie, S Barratt, P Beirne, J Blaikley, R C Chambers, N Chaudhuri, C Coleman, L Fabbri, P M George, M Gibbons, F Gleeson, B Gooptu, I Hall, N A Hanley, L P Ho, E Hufton, J Jacob, S Johnson, M Jones, S Jones, F Khan, J Mitchell, P L Molyneaux, J E Pearl, K Piper Hanley, K Poinasamy, J Quint, P Rivera-Ortega, M G Semple, J Simpson, M Spears, L G Spencer, S Stanel, I Stewart, D Thickett, R Thwaites, L V Wain, S Walker, S Walsh, J Wild, D G Wootton, L Wright

Metabolic

S Heller (*Co-Lead*), H Atkins, S Bain, M Davies, J Dennis, K Ismail, D Johnston, P Kar, K Khunti, C Langenberg, P McArdle, A McGovern, T Peto, J Petrie, E Robertson, N Sattar, K Shah, J Valabhji, B Young

Pulmonary and Systematic Vasculature

L Howard (*Co-Lead*), Mark Toshner (*Co-Lead*), C Berry, P Chowienczyk, D Lasserson, A Lawrie, O C Leavy, J Mitchell, L Price, J Quint, J Rossdale, N Sattar, C Sudlow, A A R Thompson, J M Wild, M Wilkins

Rehabilitation, Sarcopenia and Fatigue

T Chalder (*Co-Lead*), J M Lord (*Co-Lead*), N J Greening (*Co-Lead*), W Man (*Co-Lead*), S J Singh (*Co-Lead*), N Armstrong, E Baldry, N Basu, M Beadsworth, L Bishop, C E Bolton, A Briggs, M Buch, G Carson, J Cavanagh, H Chinoy, E Daynes, S Defres, R A Evans, P Greenhaff, S Greenwood, M Harvie, M Husain, A McArdle, A

McMahon, M McNarry, C Nolan, J Pimm, J Sargent, J Scott, L Sigfrid, M Steiner, D Stensel, A L Tan, J Whitney, D Wilkinson, D Wilson, M Witham, T Yates

Renal

N Brunskill (*Co-Lead*), S Francis (*Co-Lead*), S Greenwood (*Co-Lead*), C Laing (*Co-Lead*), D Thomas (*Co-Lead*), K Bramham, P Chowdhury, A Frankel, L Lightstone, S McAdoo, K McCafferty, M Ostermann, N Selby, C Sharpe, M Willicombe

Platforms

Bioresource

W Greenhalf (*Co-Lead*), M G Semple (*Co-Lead*), M Ashworth, H Hardwick, M Sereno, R Saunders, A Singapuri, V Shaw, A Shikotra, L V Wain

Data Hub

J K Baillie (*Co-Lead*), A B Docherty (*Co-Lead*), E M Harrison (*Co-Lead*), A Sheikh (*Co-Lead*), C E Brightling, L Daines, S Dunn, R A Evans, S Kerr, O C Leavy, N I Lone, H McAuley, R Pius, M Richardson, M Sereno, L V Wain

Imaging Alliance

C Bloomfield (*Co-Lead*), M Halling-Brown (*Co-Lead*), F Gleeson (*Co-Lead*), J Jacob (*Co-Lead*), S Neubauer (*Co-Lead*) B Raman (*Co-Lead*) S Siddiqui (*Co-Lead*) J Wild (*Co-Lead*), P Jezzard, H Lamlum, E Tunnicliffe

Omics

L V Wain (*Co-Lead)*, J K Baillie (*Co-Lead*), H Baxendale, C E Brightling, M Brown, J D Chalmers, R A Evans, B Gooptu, W Greenhalf, H Hardwick, R G Jenkins, D Jones, I Koychev, C Langenberg, A Lawrie, P L Molyneaux, A Shikotra, J Pearl, M Ralser, N Sattar, R Saunders, J Scott, T Shaw, D Thomas, D Wilkinson

PHOSP-COVID Study Central Coordinating Team

C E Brightling (Chief Investigator), R A Evans (*Lead Co-I*), L V Wain (*Lead Co-I*), S Diver, R Dowling, C Edwardson, O Elneima, S Finney, R Free, N J Greening, B Hargadon, V Harris, L Houchen, W Ibrahim, O C Leavy, A Ient, H McAuley, P Novotny, C Overton, T Plekhanova, R Saunders, M Sereno, A Singapuri, M Sharma, A Shikotra, C Taylor, S Terry, E Turner, C Tong, A J Yousuf, B Zhao

Local Clinical Centre PHOSP-COVID trial staff

(listed in alphabetical order)

Aneurin Bevan University Health Board

S Fairbairn (PI), A Dell, N Hawkings, J Haworth, M Hoare, A Lucey, V Lewis, G Mallison, H Nassa, C Pennington, A Price, C Price, A Storrie, G Willis, S Young

Barts Health NHS Trust & Queen Mary University of London

P Pfeffer (PI), K Chong-James, C David, W Y James, A Martineau, O Zongo

Belfast Health and Social Care Trust & Queen's University Belfast

L G Heaney (PI), C Armour, V Brown, T Craig, S Drain, B King, N Magee, D McAulay, E Major, L McGarvey, J McGinness, R Stone

Betsi Cadwaladr University Health Board

A Haggar (PI), A Bolger, F Davies, J Lewis, A Lloyd, E McIvor, D Menzies, W Saxon, D Southern, C Subbe, V Whitehead

Bradford Teaching Hospitals NHS Foundation Trust

D Saralaya (PI), L Brear, K Regan, K Storton

Cambridge University NHS Foundation Trust & University of Cambridge

J Fuld (PI), I Cruz, K Dempsey, A Elmer, H Jones, S Jose, S Marciniak, M Parkes, M Toshner, L Watson, J Worsley

Cardiff and Vale University Health Board

R Sabit (PI), L Broad, A Buttress, T Evans, L Knibbs, A McQueen, C Oliver, K Paradowski, J Williams

Guy’s and St Thomas’ NHS Foundation Trust

N Hart (PI), F Adeyemi, G Arbane, S Betts, A Dewar, G Kaltsakas

Hull University Teaching Hospitals NHS Trust & University of Hull

N Easom (PI), P Atkin, K Brindle, M G Crooks, R Flockton, L Holdsworth, A Richards, D L Sykes, S Thackray-Nocera, K Vellore, C Wright

Hywel Dda University Health Board

M Andrews (PI), K E Lewis (PI), A Mohamed (PI), G Ross (PI), K Davies, R Hughes, R Loosley, L O'Brien, Z Omar, H McGuinness, E Perkins, J Phipps, A Taylor, H Tench, R Wolf-Roberts

Imperial College Healthcare NHS Trust & Imperial College London

L Howard (PI), O Kon (PI), D C Thomas (PI), B Card, E Calvelo, E R Chilvers, P Cullinan, R G Jenkins, C King, M Mariveles, U Munawar, J Nunag, R Parvin, B Pathmanathan, E Russell

King's College Hospital NHS Foundation Trust & Kings College London

A Shah (PI), C J Jolley (PI), O Adeyemi, H Assefa-Kebede, J Breeze, M Brown, S Byrne, T Chalder, P Dulawan, N Hart, A Hayday, A Hoare, A Knighton, M Malim, S Patale, I Peralta, N Powell, A Ramos, A Shah, K Shevket, F Speranza, A Te

Leeds Teaching Hospitals & University of Leeds

P Beirne (PI), A Ashworth, J Clarke, C Coupland, M Dalton, E Wade, C Favager, J Greenwood, J Glossop, L Hall, A Humphries, J Murira, D Peckham, S Plein, J Rangeley, A L Tan, B Whittam, N Window, J Woods

Liverpool University Hospitals NHS Foundation Trust & University of Liverpool

D G Wootton (PI), L Turtle (PI), J Brown, A Cross, S L Dobson, N French, W Greenhalf, H Hardwick, K Hainey, J Hawkes, AL Key, N Lewis-Burke, G Madzamba, M J Noonan, L Poll, M G Semple, V Shaw, K A Tripp, L O Wajero, S A Williams-Howard

London North West University Healthcare NHS Trust

S N Diwanji (PI), P Papineni (PI), S Gurram, S Quaid, G F Toingson, E Watson

Manchester University NHS Foundation Trust & University of Manchester

B Al-Sheklly (PI), A Horsley (PI), C Avram, J Blaikely, M Buch, N Choudhury, D Faluyi, T Felton, T Gorsuch, N A Hanley, T Hussell, Z Kausar, N Odell, R Osbourne, K Piper Hanley

Newcastle upon Tyne Hospitals NHS Foundation Trust & University of Newcastle

A De Soyza (PI), C Echevarria (PI), J Brown, G Burns, G Davies, H Fisher, C Francis, A Greenhalgh, P Hogarth, J Hughes, K Jiwa, G Jones, G MacGowan, D Price, A Sayer, J Simpson, H Tedd, S West, M Witham, S Wright, A Young

NHS Dumfries and Galloway

M J McMahon (PI), P Neill

NHS Greater Glasgow and Clyde Health Board & University of Glasgow

D Anderson (PI), H Bayes (PI), C Berry (PI), D Grieve (PI), I B McInnes (PI), N Basu, A Brown, A Dougherty, K Fallon, L Gilmour, K Mangion, A Morrow, K Scott, R Sykes

NHS Highland

E K Sage (PI), F Barrett, A Donaldson

NHS Lanarkshire

M Patel (PI), D Bell, A Brown, M Brown, R Hamil, K Leitch, L Macliver, J Quigley, A Smith, B Welsh

NHS Lothian & University of Edinburgh

G Choudhury (PI), J K Baillie, S Clohisey, A Deans, A B Docherty, J Furniss, E M Harrison, S Kelly, N I Lone, A Sheikh

NHS Tayside & University of Dundee

J D Chalmers (PI), D Connell, J George, C J Tee, J Rowland, D Sutherland, A Elliott

North Bristol NHS Trust & University of Bristol

N Maskell (PI), D Arnold, S Barrett, H Adamali, A Dipper, S Dunn, A Morley, L Morrison, L Stadon, H Welch

North Middlesex Hospital NHS Trust

B Jayaraman (PI), T Light

Nottingham University Hospitals NHS Trust & University of Nottingham

C E Bolton (PI), J Bonnington, C Dobson, P Greenhaff, A Gupta, L Howard, S Linford, L Matthews, A K Thomas

Oxford University Hospitals NHS Foundation Trust & University of Oxford

L P Ho (PI), N M Rahman (PI), M Ainsworth, A Alamoudi, A Bloss, P Carter, J Chen, T Dong, R I Evans, E Fraser, J R Geddes, F Gleeson, P Harrison, M Havinden-Williams, P Jezzard, I Koychev, P Kurupati, H McShane, S Neubauer, D Nicoll, G Ogg, E Pacpaco, M Pavlides, Y Peng, N Petousi, N Rahman, B Raman, M J Rowland, K Saunders, M Sharpe, N Talbot, E Tunnicliffe

Royal Brompton and Harefield NHS Foundation Trust

W Man (PI), B Patel (PI), R E Barker, D Cristiano, N Dormand, M Gummadi, S Kon, K Liyanage, C M Nolan, Patel, O Polgar, P Shah, S J Singh, J A Walsh

Royal Free London NHS Foundation Trust

J Hurst (PI), H Jarvis (PI), S Mandal (PI), S Ahmad, S Brill, L Lim, D Matila, E Murali, O Olaosebikan, C Singh

Royal Papworth Hospital NHS Foundation Trust

M Toshner (PI), H Baxendale, L Garner, C Johnson, A Michael, H Parfrey, J Parmar

Salford Royal NHS Foundation Trust

N Diar Bakerly (PI), A Atkins, P Dark, D Evans, E Gourlay, E Hardy, A Harvey, D Holgate, S Knight, N Mairs, N Majeed, L McMorrow, J Oxton, J Pendlebury, C Summersgill, R Ugwuoke, S Whittaker

Sheffield Teaching NHS Foundation Trust & University of Sheffield

S Rowland-Jones (PI), A Alli, A Angyal, L Armstrong, M Bayley, E Bradley, R Brown, K Chapman, L Chetham, C Clark, Z Coburn, J Cole, T De Silva, M Dixon, A Fairman, D Foote, A Ford, R Gregory, K Harrington, S Heller, L Hesselden, A Holbourn, B Holroyd-Hind, A Howell, A Lawrie, E Lee, R Lenagh, I Macharia, S Megson, J Meiring, H Newell, J Rodgers, I Smith, J Smith, L Smith, A A R Thompson, H Turton, J Watson, L Watson, J Wild, I Wilson, A Zawia

St George's University Hospitals NHS Foundation Trust

R Aul (PI), M Ali, A Dunleavy, D Forton, N Msimanga, M Mencias, S Siddique, V Tavoukjian

Swansea Bay University Health Board

G A Davies (PI), L Connor, A Cook, T Rees, F Thaivalappil

The Hillingdon Hospitals NHS Foundation Trust

S S Kon (PI), H Lota, G Landers, S Portukhay

University College London Hospital & University College London

M Marks (PI), J S Brown (PI), R C Chambers, A Checkley, R Evans, M Heightman, T Hillman, J Hurst, J Jacob, S Janes, M Lipman, S Logan, D Lomas, J Porter, K Roy, E Wall

University Hospital Birmingham NHS Foundation Trust & University of Birmingham

D Parekh (PI), R Baggott, A Botkai, P Clift, B Cooper, J Dasgin, N Gautam, N Ghatum, T Hiwot, T Jackson, S Johnson, V Kamwa, J M Lord, S Madathil, A Newton Cox, J Nyaboko, H Qureshi, E Sapey, J Short, J Stockley, Z Suleiman, T Thompson, S Walder, C Welch, D Wilson, S Yasmin, K P Yip

University Hospitals of Leicester NHS Trust & University of Leicester

C E Brightling (CI), R A Evans (PI), M Aljaroof, N Armstrong, H Arnold, M Bakali, M Bakau, M Bourne, C Bourne, N Brunskill, P Cairns, L Carr, A Charalambou, C Christie, M Davies, S Diver, S Edwards, C Edwardson, O Elneima, H Evans, J Finch, S Glover, N Goodman, B Gootpu, N J Greening, K Hadley, P Haldar, B Hargadon, L Houchen, W Ibrahim, L Ingram, K Khunti, A Lea, D Lee, G P McCann, H McCauley, P McCourt, T Mcnally, A Moss, W Monteiro, M Pareek, S Parker, A Rowland, A Prickett, R Russell, J Skeemer, S Siddiqui, S J Singh, M Soares, E Stringer, T Thornton, M Tobin, L V Wain, F Woodhead, T Yates, A Yousuf

University Hospital Southampton NHS Foundation Trust & University of Southampton

M Jones (PI), R Djukanovic, S Fletcher, M Harvey, B Marshall, R Samuel, T Sass, T Wallis, H Wheeler

Whittington Health NHS

R Dharmagunawardena (PI), E Bright, P Crisp, M Stern

Health and Care Research Wales

Y Ellis

London School of Hygiene & Tropical Medicine (LSHTM)

M Marks, A Briggs

NIHR Office for Clinical Research Infrastructure

K Holmes

Patient Public Involvement Leads

Asthma UK and British Lung Foundation Partnership - K Poinasamy, S Walker

Royal Surrey NHS Foundation Trust

M Halling-Brown

South London and Maudsley NHS Foundation Trust & Kings College London

G Breen, M Hotopf

Swansea University & Swansea Welsh Network

K Lewis, N Williams

Supplementary Methods

Table SM1. Tier 2 outcome measures

Module	Tier 2 outcome measures reported in the current analysis	Other Tier 2 outcome measures not analysed
**Symptoms**	Patient symptom questionnaire (PHOSP-COVID study specific questionnaire)<br>Dyspnoea12 Questionnaire<br>Fatigue scale Questionnaire (FACIT)<br>Brief Pain Inventory Questionnaire (BPI)	MRC dyspnoea scale grade<br>Nottingham activities of daily living Questionnaire
**Health-related Quality of life and Disability**	Euroqol EQ5D-5L<br>Washington Short Set of Functioning	
**Respiratory**	Pulmonary Function Tests Including: Spirometry (FEV_1, FVC) and Transfer Factor (TLCO, KCO)	
**Cardiac**	Blood tests: BNP / NT-Pro-BNP	ECG Image Collection<br>Blood tests: Lipid Profile<br>Troponin I/Troponin T
**Renal**	Blood tests: eGFR	Urine tests: Albumin:Creatinine Ratio, Protein:Creatinine Ratio, Bedside urinalysis
**Pre-diabetes/diabetes**	Blood tests: HbA1C levels	
**Haematological**	Blood tests: D Dimer	Blood tests: Full Blood Count, INR, Ferritin
**Systemic inflammation**	Blood test: CRP	Blood tests: Fibrinogen
**Other organ function**		Blood tests: Liver function tests, 25-Hydroxyvitamin D, Bone Profile
**Physical performance**	Incremental Shuttle Walk Test (ISWT) to assess exercise capacity<br>Short Physical Performance Battery (SPPB)	Daily physical activity by wearable technology (Geneactive)<br>Handgrip Strength<br>General Practice Physical Activity Questionnaire (GPPAQ)
**Frailty**	Rockwood Clinical Frailty Scale (CFS)	Fried's frailty definition<br>SARC-F Questionnaire
**Body composition**	Body Mass Index (BMI) calculation from Height and Weight Measurement	Body composition estimation via: Bio-Electrical Impedance Analysis (BIA) or Duel Energy X-ray Analysis (DXA)<br>Waist Circumference Measurement
**Mental Health**	Generalised Anxiety Disorder Questionnaire (GAD-7)<br>Patient Health Questionnaire (PHQ-9)<br>Post Traumatic Stress Disorder Checklist for DSM-5 Questionnaire (PCL-5)	
**Cognition**	Montreal Cognitive Assessment (MoCA)	

FEV_1 = Forced Expiratory Volume in 1 second, FVC = Forced Vital Capacity, TLCO = Transfer Capacity of the Lung for Carbon Monoxide, KCO = carbon monoxide transfer coefficient, BNP = Brain Natriuretic Peptide or NT-BNP N-Terminal Brain Natriuretic Peptide, HbA1C = glycosylated haemoglobin, eGFR = estimated Glomerular Filtration Rate, CRP = C-Reactive Protein

Table SM2. Methods and thresholds for processing of variables and outcome measures used in the current analysis

	**Method**
**Table 1**	
Indices of Multiple Deprivation	Obtained using postcode[1]
Comorbidities	A pre-existing comorbidity was considered absent if not indicated by a 'yes' on the case report form.
Admission duration	Calculated using the hospital discharge date and the earliest admission date to the same or different hospital for the participant's COVID-19 episode.
**Table 2**	
Recovered from COVID-19?	Participants were asked: "Do you feel fully recovered from COVID-19?"<br><br>Possible answers were "Yes", "No" or "Unsure".
Symptoms at 2 to 7 months	The total number of current symptoms reported were from the following list which were answered as binary Yes/No questions: Aching in your muscles (pain), Physical slowing down, Slowing down in your thinking, Joint pain or swelling, Limb weakness, Difficulty with concentration, Short term memory loss, Headache, Tingling feeling/pins and needles, Confusion/fuzzy head, Dizziness or light headedness, Chest tightness, Problems with balance, Altered personality/ behaviour, Chest pain, Palpitations, Leg/ankle swelling, Difficulty with communication, Skin rash, Diarrhoea, Problems seeing, Pain on breathing, Weight loss, Tremor/shakiness, Constipation, Erectile Dysfunction, Loss of sense of smell, Can't fully move or control movement, Abdominal pain, Stomach pain, Loss of control of passing urine, Loss of appetite, Loss of taste, Nausea/vomiting, Bleeding, Can't move and/or feel one side of your body or face, Loss of control of opening bowels, Lumpy lesions on toes, Fainting / blackouts, Seizures<br><br>Symptom severity was rated using a 0-10 visual analogue scale for Breathlessness, Cough, Fatigue, Sleep quality and Pain before COVID-19 illness and worst in last 24 hours. The results were dichotomised using cut off of ≤ 2 for no and ≥ 3 for Yes to combine the analysis with the longer list of symptoms. For the analysis shown in Table SR6 section a) a lower score by 1 point was reported as patient worsened.
Generalised Anxiety Disorder Questionnaire (GAD-7) (Anxiety)	The Generalised Anxiety Disorder (GAD-7) questionnaire is a patient reported outcome measure consists of 7 questions with total scores ranging from 0 to 21. We used a GAD7 threshold score of > 8 to suggest at least mild-moderate anxiety.[2]
Patient Health Questionnaire (PHQ-9) (Depression)	The Patient Health Questionnaire (PHQ-9) is a patient reported outcome measure consisting of 9 questions with total scores ranging from 0 to 27. We used a PHQ-9 threshold score of ≥ 10 to suggest at least moderate depression.[3]
Post-Traumatic Stress Disorder Checklist for DSM V (PCL-5) Questionnaire	The Post-Traumatic Stress Disorder Checklist for DSM V (PCL-5) questionnaire is a patient reported outcome measure consisting of 20 questions assessing evidence of post-traumatic stress disorder according to the DSM V criteria. Total scores range from 0-80. We used a PCL-5 threshold score of ≥ 38 suggestive of a provisional diagnosis of post-traumatic stress disorder.[4,5]
Dyspnoea-12	The Dyspnoea-12 questionnaire is a patient reported outcome measure consisting of 12 questions assessing breathlessness severity incorporating both "physical" and "affective" aspects.[6] Scores range from 0 to 36 with higher scores correspond to greater severity of breathlessness.
FACIT fatigue subscale score (FACIT)	The Functional Assessment of Chronic Illness Therapy – Fatigue (FACIT-Fatigue) scale is a patient reported outcome measure consisting of 13 questions to assess self-reported fatigue and its impact on daily activities and function.[7] Total scores range from 0-52, with lower scores corresponding to an increased burden of fatigue.[8]
Brief Pain Inventory (BPI) severity and interference	The Brief Pain Inventory (BPI) is a patient reported outcome questionnaire consisting of 15 questions across domains of pain severity and pain interference. We have reported the BPI Severity score as the

	mean score from the 4 severity questions each with a range 0 – 10 anchored at 0 = "No Pain" and 10 = "Pain as bad as you can imagine".[9,10]
Short Physical Performance Battery (SPPB)	The Short Physical Performance Battery (SPPB) test is a researcher administer assessment of physical performance and frailty. It comprises 3 components; balance, gait speed and sit to stand tests. Tests were completed according to recommended standards and training was provided to site staff by the central study team via a recorded demonstration video. SPPB total scores range from 0-12. We have reported a total SPPB score of ≤10 suggestive of underlying frailty.[11-13]
Incremental Shuttle Walk Test (ISWT)	The Incremental Shuttle Walk Test (ISWT) is a researcher administered assessment of maximal physical performance and was performed according to standardised instructions with two attempts performed by participants on the same day with a 20 minutes rest between them.[14] Training was provided to site staff by the central study team via a recorded demonstration video. The best effort was reported in metres and the percent predicted value was calculated using the following reference formula accounting for gender, age and BMI.[15] ($ISWTpred = 1449{\cdot}701 - (11{\cdot}735 \times age) + (241{\cdot}897 \times gender) - (5{\cdot}686 \times BMI)$, where male gender = 1 and female gender = 0)
Rockwood Clinical Frailty Scale (CFS)	The Rockwood Clinical Frailty Scale (CFS) is a researcher assessed scale of clinical frailty with scores ranging from 1-9 where lower scores correspond to increased frailty. We have reported CFS scores of <5 suggestive of frailty.[16]
Montreal Cognitive Assessment (MoCA)	The Montreal Cognitive Assessment (MoCA) is a researcher administered cognitive function questionnaire across 8 domains. Training was provided to site staff using standardised resources supplied online by MoCA TEST Inc.[17] The assessment was conducted in English with researchers applying their discretion to exclude participants whose command of English was insufficient to complete the test accurately. Total scores range from 0 to 30. We report total MoCA scores of <23 suggestive of at least Mild Cognitive Impairment.[18]
Spirometry and Pulmonary Function Testing	Due to COVID-19 related restrictions on aerosol-generating procedures during the study period, access to spirometry and lung function was limited. Spirometry and Pulmonary function testing was completed as per ERS/ATS recommendations.[19] Spirometry and Transfer factor values were converted to SI units if not reported as such by sites. Transfer Capacity of the Lung for the uptake of carbon monoxide (TLCO) and carbon monoxide transfer coefficient (KCO) were obtained from the best of two repeat readings. ERS Reference values were used to calculate % predicted values.[20-22] $FEV_1/FVC <0{\cdot}7$ was used to define airflow obstruction.[23] % predicted TLCO <80% was considered indicative of impaired gas transfer.
BNP / NT-pro BNP	Brain Natriuretic Peptide (BNP) or N-terminal pro B-type Natriuretic Peptide (NT-pro BNP) were collected by according to each site's routine clinically available assay as a biomarker of heart failure. Three sites submitted BNP results with all of the remaining sites submitting NT-pro BNP results. The threshold values used for BNP was ≥ 100 ng/litre[24] and for NT-pro BNP ≥ 400ng/litre [25] as suggestive of heart failure.
Glycated haemoglobin (HbA1c)	Glycated haemoglobin (HbA1c) was collected as a biomarker of current glycaemic control. We have reported HbA1c levels ≥ 6·5% as suggestive of a diagnosis of diabetes.[26]
D-Dimer	D-Dimer levels were collected as a biomarker of possible thromboembolic disease with sites reporting results either as mcg/mL Fibrinogen Equivalent Unit (FEU) or ng/mL D Dimer Unit (DDU) according to their clinically available assays. Conversion of mcg/mL FEU to ng/mL DDU used the following equation: (value in mcg/mL FEU)*500<br><br>We have reported a D-Dimer level ≥ 500ng/ml as suggestive of systemic inflammation and possible venous thromboembolic disease.[27]
C-Reactive Protein (CRP)	C-Reactive Protein (CRP) levels were collected as a biomarker of current systemic inflammation. Values reported as below the lower or upper limit reportable range for the assay used at the site have been included at the stated less than or more than cut off value for calculation of mean (SD) results. We have reported CRP levels > 10mg/L as suggestive of systemic inflammation.
**Table SR8**	
EQ5D-5L VAS	The EQ5D Visual Analogue Scale is a patient reported outcome questionnaire recording the patient's self-rated health and was completed for "before your COVID-19 illness" and "your own health state today." Scores are presented as mean and standard deviation.[28]
EQ5D-5L Utility Index	The EQ5D-5L is a five-dimension patient reported outcome questionnaire recording a patient's self-rated health state for mobility, self-care, usual activities, pain/discomfort and anxiety/depression. These scores

	are then mapped to a United Kingdom specific Utility Index anchored at 1 for "perfect health" and 0 for "dead" calculated from reported EQ5D-5L scores across the five dimensions.[29]
Washington Group Short Set of Functioning	The Washington Group Short Set of Functioning (WG-SS) is a patient reported outcome questionnaire using six questions to assess disability and function. A participant was considered to have a new disability if a response for any single domain changed from "no difficulty" or "some difficulty" to "a lot of difficulty" or "cannot do it at all" following the Washington Group guidelines.[30]

Supplementary Data – Results

Table SR1. Co-morbidities for the cohort stratified by severity of acute illness using the WHO clinical progression scale

	WHO – class 3-4	WHO – class 5	WHO – class 6	WHO – class 7-9	Total
Total N (%)	226 (21·0)	378 (35·1)	185 (17·2)	288 (26·7)	1077
**CARDIOVASCULAR DISEASE**	··	··	··	··	··
Myocardial Infarction	11 (5·0)	20 (5·5)	3 (1·7)	7 (2·5)	41 (3·9)
Ischaemic Heart Disease	14 (6·4)	27 (7·4)	5 (2·9)	16 (5·7)	62 (6·0)
Atrial Fibrillation	10 (4·5)	17 (4·7)	14 (8·1)	7 (2·5)	48 (4·6)
Hypertension	56 (25·5)	126 (34·5)	62 (35·8)	110 (39·3)	354 (34·1)
Congestive Heart Failure	4 (1·8)	5 (1·4)	2 (1·2)	4 (1·4)	15 (1·4)
Congenital Heart Disease	0 (0·0)	2 (0·5)	1 (0·6)	2 (0·7)	5 (0·5)
Valvular Heart Disease	1 (0·5)	6 (1·6)	5 (2·9)	2 (0·7)	14 (1·3)
Pacemaker / Implantable Defibrillator	6 (2·7)	5 (1·4)	1 (0·6)	2 (0·7)	14 (1·3)
Peripheral Vascular Disease	4 (1·8)	5 (1·4)	1 (0·6)	2 (0·7)	12 (1·2)
Hypercholesterolaemia/dyslipidaemia	25 (11·4)	74 (20·3)	39 (22·7)	45 (16·1)	183 (17·7)
Cerebrovascular Accident/ Transient Ischaemic Attack	8 (3·7)	23 (6·4)	7 (4·1)	13 (4·7)	51 (4·9)
**NEUROLOGICAL and PSYCHIATRIC**	··	··	··	··	··
Dementia	0 (0·0)	4 (1·1)	0 (0·0)	0 (0·0)	4 (0·4)
Depression or Anxiety	32 (14·6)	45 (12·3)	29 (16·8)	40 (14·3)	146 (14·1)
Chronic Fatigue Syndrome/fibromyalgia or chronic pain	10 (4·6)	11 (3·0)	5 (2·9)	12 (4·3)	38 (3·7)
Previous treatment with antidepressant medication	21 (9·6)	34 (9·3)	23 (13·4)	24 (8·6)	102 (9·9)
Previous treatment with a mental health professional for a mental health problem	12 (5·5)	14 (3·8)	13 (7·6)	13 (4·7)	52 (5·0)
**RESPIRATORY**	··	··	··	··	··
COPD	10 (4·6)	23 (6·3)	11 (6·4)	5 (1·8)	49 (4·7)
Asthma	41 (18·6)	64 (17·6)	36 (20·8)	47 (16·8)	188 (18·1)
Interstitial Lung Disease	1 (0·5)	3 (0·8)	1 (0·6)	3 (1·1)	8 (0·8)
Bronchiectasis	7 (3·2)	5 (1·4)	4 (2·3)	3 (1·1)	19 (1·8)
Obstructive Sleep Apnoea	8 (3·6)	20 (5·5)	11 (6·4)	14 (5·0)	53 (5·1)
Obesity hypoventilation syndrome	0 (0·0)	0 (0·0)	2 (1·2)	2 (0·7)	4 (0·4)
Pleural Effusion	1 (0·5)	7 (1·9)	0 (0·0)	2 (0·7)	10 (1·0)
**RHEUMATOLOGICAL**	··	··	··	··	··
Connective Tissue Disease	1 (0·5)	0 (0·0)	0 (0·0)	0 (0·0)	1 (0·1)

Rheumatoid Arthritis	8 (3·6)	9 (2·5)	5 (2·9)	5 (1·8)	27 (2·6)
Osteoarthritis	18 (8·2)	39 (10·7)	14 (8·1)	25 (8·9)	96 (9·3)
**GASTROINTESTINAL**	··	··	··	··	··
Peptic Ulcer Disease	2 (0·9)	4 (1·1)	0 (0·0)	2 (0·7)	8 (0·8)
Liver disease - *Mild*	4 (1·8)	4 (1·1)	5 (2·9)	3 (1·1)	16 (1·5)
Liver disease – *mod/severe*	5 (2·3)	3 (0·8)	5 (2·9)	4 (1·4)	17 (1·6)
GORD	23 (10·5)	38 (10·4)	15 (8·7)	27 (9·7)	103 (10·0)
Inflammatory Bowel Disease	3 (1·4)	6 (1·6)	2 (1·2)	2 (0·7)	13 (1·3)
Irritable Bowel Disease	9 (4·1)	3 (0·8)	7 (4·1)	7 (2·5)	26 (2·5)
**METABOLIC/ENDOCRINE/RENAL**	··	··	··	··	··
Diabetes *Type 1*	1 (0·5)	6 (1·6)	0 (0·0)	1 (0·4)	8 (0·8)
Diabetes *Type 2*	30 (13·7)	80 (21·9)	40 (23·1)	63 (22·7)	213 (20·6)
Uncomplicated (% of all Diabetes)	*21 (67·7)*	*58 (67·4)*	*25 (62·5)*	*39 (60·9)*	*143 (64·7)*
End-organ damage (% of all Diabetes)	*0 (0·0)*	*2 (2·3)*	*2 (5)*	*3 (4·7)*	*7 (3·2)*
Hypothyroidism	15 (6·8)	16 (4·4)	2 (1·2)	14 (5·0)	47 (4·5)
Hyperthyroidism	1 (0·5)	9 (2·5)	1 (0·6)	3 (1·1)	14 (1·3)
Chronic kidney disease	8 (3·6)	21 (5·8)	8 (4·6)	14 (5·0)	51 (4·9)
**MALIGNANCY**	··	··	··	··	··
Solid tumour malignancy	··	··	··	··	··
Localised	5 (2·3)	19 (5·2)	5 (2·9)	4 (1·4)	33 (3·2)
Metastatic	2 (0·9)	3 (0·8)	0 (0·0)	2 (0·7)	7 (0·7)
Leukaemia	1 (0·5)	5 (1·4)	2 (1·2)	3 (1·1)	11 (1·1)
Lymphoma	4 (1·8)	4 (1·1)	2 (1·2)	1 (0·4)	11 (1·1)
**CHRONIC INFECTIOUS DISEASE**	··	··	··	··	··
HIV	0 (0·0)	1 (0·3)	1 (0·6)	3 (1·1)	5 (0·5)
Chronic Viral Hepatitis (B or C)	5 (2·3)	1 (0·3)	3 (1·7)	4 (1·5)	13 (1·3)
Mycobacterium TB (previously treated active or latent)	5 (2·3)	3 (0·8)	2 (1·2)	3 (1·1)	13 (1·3)

Data are n (%). WHO = World Health Organisation. Category 3-4 = no continuous supplemental oxygen needed, 5= continuous supplemental oxygen only, 6= Continuous or Bi-level Positive Airway Pressure ventilation or High Flow Nasal O_2, 7-9 = Invasive Mechanical Ventilation or other organ support

Table SR2. Occupation data at baseline, and change in employment by severity of acute illness and by cluster severity

Table SR2a. Occupation data at before hospitalisation for COVID-19 stratified by severity of acute illness

	**WHO – class 3-4**	**WHO – class 5**	**WHO – class 6**	**WHO – class 7-9**	**Total**
Working full-time	110 (54·5)	172 (52·6)	90 (53·9)	175 (68·9)	547 (57·6)
Working part-time	23 (11·4)	31 (9·5)	19 (11·4)	21 (8·3)	94 (9·9)
Full time carer (children or other)	5 (2·5)	6 (1·8)	0 (0·0)	0 (0·0)	11 (1·2)
Unemployed	9 (4·5)	11 (3·4)	2 (1·2)	6 (2·4)	28 (2·9)
Unable to work due to chronic illness	3 (1·5)	6 (1·8)	4 (2·4)	7 (2·8)	20 (2·1)
Student	3 (1·5)	2 (0·6)	2 (1·2)	3 (1·2)	10 (1·1)
Retired	43 (21·3)	95 (29·1)	47 (28·1)	39 (15·4)	224 (23·6)
Medically retired	5 (2·5)	2 (0·6)	3 (1·8)	3 (1·2)	13 (1·4)
Prefer not to say	1 (0·5)	2 (0·6)	0 (0·0)	0 (0·0)	3 (0·3)
(Missing)	24	51	18	34	127

Variables are presented as n and % of total n in each severity category. % are out of a total of 950. WHO = World Health Organisation. Category 3-4 = no continuous supplemental oxygen needed, 5= continuous supplemental oxygen only, 6= Continuous or Bi-level Positive Airway Pressure ventilation or High Flow Nasal O_2, 7-9 = Invasive Mechanical Ventilation or other organ support

Table SR2b. Change in occupation status COVID stratified by severity of acute illness

	**WHO – class 3-4**	**WHO – class 5**	**WHO – class 6**	**WHO – class 7-9**	**Total**
Working full-time or part-time before COVID-19	133	203	109	196	641
No longer working after COVID-19	15 (11·3)	24 (11·8)	20 (18·3)	54 (27·6)	113 (17·8)
Occupation change due to health after COVID-19	19 (14·3)	19 (9·4)	18 (16·5)	68 (34·7)	124 (19·3)

Variables are presented as n and % of total n in each severity category. Percentages are the proportion of those who reported working full-time or part-time before COVID-19. Participants were classified as no longer working post-hospitalisation for COVID-19 if they reported working full or part-time before COVID-19, subsequently answered "different from before" when asked "What is your main occupation/working status today?" and answered, "Unable to work due to chronic illness/ /Medically retired". Participants who reported working full or part-time before COVID-19 were classified as experiencing an occupation change due to health if they answered "different from before" when asked "What is your main occupation/working status today?" and then answered "Poor health/Sick leave" when asked "If different, why did your occupation/working status change?".

Table SR2c. Occupation data before hospitalisation for COVID-19 stratified by cluster

	Cluster 1 "Very Severe"	Cluster 2 "Severe"	Cluster 3 "Moderate & Cognitive"	Cluster 4 "Mild"	Total
Working full-time	59 (52·2)	94 (67·1)	45 (42·9)	211 (66·6)	409 (60·6)
Working part-time	11 (9·7)	14 (10·0)	17 (16·2)	20 (6·3)	62 (9·2)
Full time carer (children or other)	4 (3·5)	1 (0·7)	0 (0·0)	2 (0·6)	7 (1·0)
Unemployed	9 (8·0)	2 (1·4)	1 (1·0)	4 (1·3)	16 (2·4)
Unable to work due to chronic illness	8 (7·1)	3 (2·1)	0 (0·0)	2 (0·6)	13 (1·9)
Student	1 (0·9)	1 (0·7)	1 (1·0)	3 (0·9)	6 (0·9)
Retired	16 (14·2)	22 (15·7)	38 (36·2)	74 (23·3)	150 (22·2)
Medically retired	5 (4·4)	3 (2·1)	1 (1·0)	0 (0·0)	9 (1·3)
Prefer not to say	0 (0·0)	0 (0·0)	2 (1·9)	1 (0·3)	3 (0·4)
(Missing)	18	19	22	33	92

Variables are presented as n and % of total n in each severity category.

Table SR2d. Change in occupation status after hospitalisation for COVID-19 stratified by cluster.

	Cluster 1 "Very Severe"	Cluster 2 "Severe"	Cluster 3 "Moderate & Cognitive"	Cluster 4 "Mild"	Total
Working full-time or part-time before COVID-19	70	108	62	231	471
No longer working after COVID-19	35 (50·0)	12 (11·1)	10 (16·1)	23 (10·0)	78 (16·6)
Occupation change due to health after COVID-19	42 (60·0)	21 (19·4)	10 (16·1)	20 (8·7)	93 (19·7)

Variables are presented as n and % of total n in each severity category. See footnote to Table SR2b

Table SR3. Patient reported outcomes, physiological and biochemical tests stratified by severity of acute illness

	N (%)	WHO – class 3-4	WHO – class 5	WHO – class 6	WHO – class 7-9	Total	p
Total N (%)	··	226 (21·0)	378 (35·1)	185 (17·2)	288 (26·7)	1077	··
**PROMS**	··	··	··	··	··	··	··
Persistent symptom	861 (79·9)	167 (94·4)	264 (89·5)	135 (92·5)	231 (95·1)	797 (92·6)	0·070
Symptom count †	861 (79·9)	10·0 (4·0 to 19·0)	7·0 (3·0 to 13·0)	8·0 (4·0 to 16·0)	9·0 (5·0 to 16·0)	9·0 (4·0 to 16·0)	<0·001
GAD7 total score ††	1031 (95·7)	5·8 (6·2)	4·4 (5·3)	5·4 (5·7)	5·5 (6·0)	5·2 (5·8)	0·017
Anxiety (GAD7 >8)	1031 (95·7)	57 (26·8)	72 (19·9)	44 (25·3)	80 (28·4)	253 (24·5)	0·069
Missing	··	13	16	11	6	46	··
PHQ-9 total score ††	1029 (95·5)	7·6 (7·0)	5·7 (6·0)	6·4 (6·1)	7·3 (6·7)	6·7 (6·5)	0·002
Depression (PHQ-9 ≥ 10)	1029 (95·5)	64 (30·2)	79 (21·9)	49 (28·0)	90 (32·0)	282 (27·4)	0·024
Missing	··	14	17	10	7	48	··
PCL-5 Total Severity Score ††	1030 (95·6)	15·9 (18·6)	12·5 (14·7)	15·5 (17·1)	18·6 (19·1)	15·4 (17·3)	<0·001
PTSD (PCL-5 ≥38)	1030 (95·6)	29 (13·6)	31 (8·5)	21 (12·0)	45 (16·3)	126 (12·2)	0·025
Missing	··	12	13	10	12	47	··
Dyspnoea-12 score ††	1017 (94·4)	7·2 (9·4)	5·5 (7·7)	6·5 (8·8)	6·5 (8·8)	6·3 (8·6)	0·107
FACIT fatigue subscale score ††	1036 (96·2)	18·5 (14·3)	14·6 (12·1)	16·4 (13·1)	18·5 (13·4)	16·8 (13·2)	<0·001
BPI severity ††	801 (74·4)	12·7 (10·3)	12·8 (10·6)	11·6 (9·6)	13·1 (10·3)	12·7 (10·3)	0·558
BPI interference ††	777 (72·1)	19·8 (20·8)	17·6 (18·2)	15·1 (16·7)	20·4 (19·6)	18·4 (19·0)	0·059
**Body composition by BMI** kg/m^2	908 (84·3)	··	··	··	··	··	··
Underweight (<18·5)	··	2 (1·1)	2 (0·6)	1 (0·6)	1 (0·4)	6 (0·7)	0.007
Normal weight (18·5 to 24·9)	··	43 (23·5)	42 (13·0)	16 (10·1)	27 (11·1)	128 (14·1)	··
Overweight (25 to 29·9)	··	58 (31·7)	122 (37·8)	45 (28·3)	85 (35·0)	310 (34·1)	··
Obese (30 to 39·9)	··	67 (36·6)	131 (40·6)	76 (47·8)	101 (41·6)	375 (41·3)	··
Severe obesity (40+)	··	13 (7·1)	26 (8·0)	21 (13·2)	29 (11·9)	89 (9·8)	··
Missing	··	43	55	26	45	169	··
**Physical performance**	··	··	··	··	··	··	··
SPPB total score (0-12) ††	970 (90·1)	10·0 (2·3)	9·9 (2·5)	9·9 (2·5)	9·7 (2·4)	9·9 (2·4)	0·698
SPPB ≤10 (mobility disability)	970 (90·1)	93 (46·7)	153 (44·9)	68 (40·5)	134 (51·1)	448 (46·2)	0·168
ISWT Distance (m) ††	634 (58·9)	466 (270)	445 (273)	425 (255)	411 (236)	436 (260)	0·296
ISWT % predicted	634 (58·9)	50·4 (37·8)	50·1 (38·7)	44·7 (32·4)	39·4 (31·4)	46·2 (35·8)	0·010
**Frailty and Cognition**	··	··	··	··	··	··	··

	N (%)	WHO – class 3-4	WHO – class 5	WHO – class 6	WHO – class 7-9	Total	p
Rockwood clinical frailty score ≥5¶	938 (87·1)	9 (4·6)	17 (5·0)	11 (7·2)	18 (7·2)	55 (5·9)	0·502
1 = Very Fit	938 (87·1)	28 (14·4)	50 (14·7)	28 (18·3)	21 (8·4)	127 (13·5)	0·449
2 = Well	··	68 (34·9)	109 (32·0)	43 (28·1)	76 (30·5)	296 (31·6)	··
3 = Managing Well	··	58 (29·7)	116 (34·0)	54 (35·3)	92 (36·9)	320 (34·1)	··
4 = Vulnerable	··	32 (16·4)	49 (14·4)	17 (11·1)	42 (16·9)	140 (14·9)	··
5 = Mildly Frail	··	5 (2·6)	11 (3·2)	7 (4·6)	12 (4·8)	35 (3·7)	··
6 = Moderately Frail	··	4 (2·1)	5 (1·5)	4 (2·6)	6 (2·4)	19 (2·0)	··
7 = Severely Frail	··	0 (0·0)	1 (0·3)	0 (0·0)	0 (0·0)	1 (0·1)	··
8 = Very Severely Frail	··	0 (0·0)	0 (0·0)	0 (0·0)	0 (0·0)	0 (0·0)	··
9 = Terminally Ill	··	0 (0·0)	0 (0·0)	0 (0·0)	0 (0·0)	0 (0·0)	··
(Missing)	··	31	37	32	39	139	··
MoCA score ††	888 (82·5)	25·9 (3·4)	25·1 (4·4)	25·9 (2·9)	25·8 (3·9)	25·6 (3·9)	0·063
MoCA <23	888 (82·5)	25 (13·5)	66 (21·0)	19 (12·8)	40 (16·7)	150 (16·9)	0·074
MoCA Adjusted ††	888 (82·5)	26·2 (3·4)	25·5 (4·3)	26·2 (2·9)	26·0 (3·8)	25·9 (3·8)	0·099
MoCA Adjusted <23	888 (82·5)	23 (12·4)	57 (18·1)	16 (10·8)	33 (13·8)	129 (14·5)	0·130
**Lung physiology**	··	··	··	··	··	··	··
FEV_1 (L) ††	574 (53·3)	2·6 (0·7)	2·7 (0·9)	2·7 (0·8)	2·7 (0·9)	2·7 (0·8)	0·623
FEV_1 % predicted	484 (44·9)	88·6 (19·6)	89·0 (18·3)	90·9 (31·5)	85·0 (23·9)	88·0 (22·9)	0·247
FEV_1 % predicted <80%¶	484 (44·9)	26 (28·6)	43 (26·1)	23 (28·4)	58 (39·5)	150 (31·0)	0·063
FVC (L) ††	571 (53·0)	3·3 (0·9)	3·5 (1·1)	3·4 (1·0)	3·3 (1·1)	3·4 (1·0)	0·172
FVC % predicted	481 (44·7)	88·1 (19·3)	89·5 (16·4)	92·0 (36·0)	80·7 (19·8)	87·0 (22·7)	0·001
FVC % predicted <80%¶	481 (44·7)	30 (33·0)	43 (26·4)	25 (30·9)	62 (42·5)	160 (33·3)	0·026
FEV_1/FVC ††	571 (53·0)	0·8 (0·2)	0·8 (0·1)	0·8 (0·1)	0·8 (0·2)	0·8 (0·2)	0·007
FEV_1/FVC <0·7¶	571 (53·0)	13 (12·3)	27 (13·8)	15 (16·0)	6 (3·4)	61 (10·7)	0·002
TLCO mmol/KPa/min ††	194 (18·0)	7·0 (1·8)	7·1 (1·5)	7·3 (2·0)	7·0 (2·5)	7·1 (2·0)	0·952
TLCO % predicted	169 (15·7)	97·5 (16·4)	89·6 (18·9)	97·1 (39·4)	83·3 (32·8)	89·8 (28·6)	0·099
TLCO % predicted <80%¶	169 (15·7)	3 (15·8)	19 (30·2)	6 (19·4)	30 (53·6)	58 (34·3)	0·001
KCO mmol/Kpa/min/L ††	202 (18·8)	1·5 (0·3)	1·4 (0·3)	1·4 (0·2)	1·4 (0·3)	1·4 (0·3)	0·153
KCO % predicted	174 (16·2)	104·8 (15·6)	97·1 (16·4)	98·9 (14·6)	96·1 (20·4)	97·9 (17·6)	0·289
KCO % predicted <80%¶	174 (16·2)	2 (10·5)	7 (10·9)	2 (6·2)	5 (8·5)	16 (9·2)	0·887
**Biochemical Tests**	··	··	··	··	··	··	··
BNP Result (ng/L) ††	51 (4·7)	49·6 (53·9)	40·9 (63·0)	43·6 (53·3)	38·1 (39·4)	42·2 (52·0)	0·960
Pro-NT-BNP (ng/L) ††	572 (53·1)	129 (297)	173 (382)	177 (460)	305 (1832)	201 (1023)	0·473

	N (%)	WHO – class 3-4	WHO – class 5	WHO – class 6	WHO – class 7-9	Total	p
BNP/NT-Pro-BNP above threshold*¶	621 (57·7)	8 (5·8)	15 (7·2)	8 (8·0)	15 (8·5)	46 (7·4)	0·825
HbA1C % (DCCT/NGSP) ††	611 (56·7	5·9 (1·1)	6·3 (1·3)	6·1 (1·1)	6·0 (1·2)	6·1 (1·2)	0·010
HbA1C ≥6·0 ¶	611 (56·7)	37 (27·2)	90 (42·3)	39 (41·1)	47 (28·1)	213 (34·9)	0·004
eGFR Result (ml/min/1·73m2) ††	845 (78·5)	89·0 (76·6)	79·5 (44·4)	81·2 (62·6)	84·2 (83·1)	83·0 (66·7)	0·493
eGFR < 60 ml/min/1·73 m^2¶	845 (78·5)	15 (8·5)	42 (14·0)	18 (13·0)	38 (16·4)	113 (13·4)	0·138
D-Dimer Result (mg/L) ††	738 (68·5)	285·2 (430·4)	332·5 (381·6)	344·6 (369·0)	233·2 (168·8)	298·0 (349·7)	0·008
D-dimer ≥500 ng/ml¶	738 (68·5)	15 (9·7)	45 (17·2)	22 (17·6)	15 (7·6)	97 (13·1)	0·005
**Systemic Inflammation**	··	··	··	··	··	··	··
CRP (>10 mg/L)¶	804 (74·7)	18 (10·7)	24 (8·4)	13 (10·0)	35 (16·1)	90 (11·2)	0·052
CRP (>5 mg/L)¶	804 (74·7)	36 (21·3)	59 (20·6)	26 (20·0)	59 (27·1)	180 (22·4)	0·279

Missing not included in %, Number (%) unless †median [IQR], †† mean [SD], ¶ = % of category with positive response *Threshold - BNP ≥100ng/L or NT-BNP ≥400ng/L, column proportions, P values for Chi-squared test for differing proportions across WHO categories are presented, P values for Kruskal-Wallis tests for variables summarised as median (IQR) are presented and P values for ANOVA F-test for variables summarised as mean [SD] are presented. DCCT/NGSP - Diabetes Control and Complications Trial / National Glycohemoglobin Standardization Programme, PROM = Patient reported outcome measures, GAD7 = General Anxiety Disorder 7 Questionnaire, PHQ-9 = Patient Health Questionnaire-9, PCL-5 = Post Traumatic Stress Disorder Checklist, Dyspnoea-12 Questionnaire, FACIT Fatigue Scale (FACIT), BPI =Brief Pain Inventory, SPPB = Short Physical Performance Battery, ISWT = Incremental Shuttle Walking Test, CFS = Clinical Frailty Scale, MoCA = Montreal Cognitive Assessment, FEV1 = Forced Expiratory Volume in 1 second, FVC = Forced Vital Capacity, TLCO = Transfer Capacity of the Lung for Carbon Monoxide, KCO = carbon monoxide transfer coefficient, BNP = Brain Natriuretic Peptide or NT-BNP N-Terminal Brain Natriuretic Peptide, HbA1C = glycosylated haemoglobin, eGFR = estimated Glomerular Filtration Rate, CRP = C-Reactive Protein. WHO = World Health Organisation. Category 3-4 = no continuous supplemental oxygen needed, 5= continuous supplemental oxygen only, 6= Continuous or Bi-level Positive Airway Pressure ventilation or High Flow Nasal O_2, 7-9 = Invasive Mechanical Ventilation or other organ support

Table SR4. Comparison between imputed and non-imputed logistic regression of predictors of failure to recover (multi-variable and multi-level)

Dependent: 'Fully Recovered'	No n (%)	Yes n (%)	OR (univariable)	OR (multivariable)	OR (multivariable imputation)	OR (multilevel imputation)
**Age (y)**	··	··	··	··	··	··
50-59	183 (78·2)	51 (21·8)	-	-	-	-
<30	16 (61·5)	10 (38·5)	2·24 (0·93-5·18, p=0·062)	1·61 (0·50-4·83, p=0·406)	2·27 (0·83-6·21, p=0·111)	2·28 (0·83-6·29, p=0·109)
30-39	43 (69·4)	19 (30·6)	1·59 (0·84-2·93, p=0·147)	1·30 (0·59-2·79, p=0·511)	1·42 (0·72-2·80, p=0·314)	1·48 (0·73-2·97, p=0·272)
40-49	88 (75·2)	29 (24·8)	1·18 (0·70-1·98, p=0·529)	0·66 (0·32-1·32, p=0·248)	1·08 (0·63-1·83, p=0·789)	1·10 (0·64-1·88, p=0·735)
60-69	192 (72·7)	72 (27·3)	1·35 (0·89-2·04, p=0·158)	1·19 (0·70-2·03, p=0·525)	1·33 (0·85-2·08, p=0·215)	1·34 (0·85-2·12, p=0·201)
70-79	71 (59·7)	48 (40·3)	2·43 (1·50-3·93, p<0·001)	2·78 (1·49-5·22, p=0·001)	1·96 (1·09-3·53, p=0·026)	2·07 (1·13-3·80, p=0·020)
80+	16 (50·0)	16 (50·0)	3·59 (1·67-7·72, p=0·001)	4·04 (1·57-10·60, p=0·004)	2·86 (1·31-6·23, p=0·008)	3·20 (1·44-7·14, p=0·005)
**Sex at birth**	··	··	··	··	··	··
Male	372 (67·1)	182 (32·9)	··	··	··	··
Female	246 (78·3)	68 (21·7)	0·56 (0·41-0·78, p=0·001)	0·50 (0·33-0·77, p=0·002)	0·61 (0·42-0·89, p=0·012)	0·62 (0·43-0·92, p=0·017)
**Ethnicity**	··	··	··	··	··	··
White	442 (75·6)	143 (24·4)	··	··	··	··
South Asian	83 (62·9)	49 (37·1)	1·82 (1·22-2·72, p=0·003)	1·68 (0·94-2·98, p=0·076)	1·58 (1·00-2·52, p=0·052)	1·54 (0·94-2·53, p=0·085)
Black	46 (66·7)	23 (33·3)	1·55 (0·89-2·61, p=0·111)	2·27 (1·12-4·54, p=0·021)	1·82 (1·01-3·28, p=0·046)	1·83 (0·99-3·39, p=0·053)
Mixed	12 (60·0)	8 (40·0)	2·06 (0·79-5·08, p=0·121)	1·56 (0·48-4·73, p=0·439)	1·59 (0·62-4·06, p=0·333)	1·69 (0·65-4·38, p=0·279)
Other	20 (52·6)	18 (47·4)	2·78 (1·42-5·41, p=0·003)	2·82 (1·05-7·33, p=0·035)	2·69 (1·31-5·53, p=0·007)	2·71 (1·32-5·59, p=0·007)
**IMD**	··	··	··	··	··	··
1	128 (74·0)	45 (26·0)	··	··	··	··
2	129 (68·3)	60 (31·7)	1·32 (0·84-2·10, p=0·230)	1·42 (0·78-2·58, p=0·252)	1·17 (0·72-1·91, p=0·521)	1·17 (0·72-1·90, p=0·522)
3	129 (75·4)	42 (24·6)	0·93 (0·57-1·51, p=0·757)	0·94 (0·50-1·76, p=0·845)	0·88 (0·52-1·50, p=0·637)	0·85 (0·50-1·45, p=0·550)
4	109 (69·0)	49 (31·0)	1·28 (0·79-2·07, p=0·314)	1·31 (0·69-2·46, p=0·408)	1·24 (0·75-2·04, p=0·397)	1·22 (0·73-2·02, p=0·450)
5	112 (70·9)	46 (29·1)	1·17 (0·72-1·90, p=0·528)	1·46 (0·78-2·75, p=0·234)	1·21 (0·73-1·99, p=0·461)	1·20 (0·73-1·99, p=0·472)
**No. of comorbidities¶**	··	··	··	··	··	··
0	150 (65·2)	80 (34·8)	··	··	··	··
1	123 (67·6)	59 (32·4)	0·90 (0·59-1·36, p=0·614)	0·85 (0·49-1·47, p=0·557)	0·99 (0·64-1·52, p=0·964)	0·97 (0·62-1·51, p=0·892)
2+	345 (75·7)	111 (24·3)	0·60 (0·43-0·85, p=0·004)	0·44 (0·27-0·73, p=0·001)	0·65 (0·45-0·95, p=0·026)	0·65 (0·44-0·95, p=0·026)
**BMI¶**	··	··	··	··	··	··
BMI <30 kg/m^2	242 (64·9)	131 (35·1)	··	··	··	··
BMI ≥30 kg/m^2	296 (79·1)	78 (20·9)	0·49 (0·35-0·67, p<0·001)	0·65 (0·43-0·97, p=0·035)	0·74 (0·54-1·03, p=0·073)	0·74 (0·53-1·04, p=0·082)
**WHO Class¶**	··	··	··	··	··	··
3-4	120 (68·2)	56 (31·8)	··	··	··	··
5	185 (63·4)	107 (36·6)	1·24 (0·84-1·85, p=0·289)	1·04 (0·59-1·85, p=0·895)	1·15 (0·74-1·79, p=0·540)	1·11 (0·71-1·76, p=0·640)

6	108 (72·5)	41 (27·5)	0·81 (0·50-1·31, p=0·399)	0·58 (0·29-1·15, p=0·120)	0·82 (0·45-1·48, p=0·500)	0·79 (0·43-1·45, p=0·436)
7-9	205 (81·7)	46 (18·3)	0·48 (0·31-0·75, p=0·001)	0·27 (0·13-0·57, p=0·001)	0·53 (0·30-0·94, p=0·029)	0·54 (0·30-0·96, p=0·034)
**Steroids**¶	··	··	··	··	··	··
No	376 (69·1)	168 (30·9)	··	··	··	··
Yes	181 (73·6)	65 (26·4)	0·80 (0·57-1·12, p=0·204)	1·22 (0·77-1·94, p=0·394)	1·00 (0·69-1·44, p=0·994)	1·02 (0·70-1·49, p=0·909)
**Antibiotics**¶	··	··	··	··	··	··
No	114 (72·6)	43 (27·4)	··	··	··	··
Yes	481 (71·0)	196 (29·0)	1·08 (0·74-1·61, p=0·696)	1·49 (0·87-2·60, p=0·149)	1·20 (0·77-1·87, p=0·416)	1·21 (0·77-1·90, p=0·410)
**Anticoagulation** ¶	··	··	··	··	··	··
No	353 (67·4)	171 (32·6)	··	··	··	··
Yes	207 (75·3)	68 (24·7)	0·68 (0·49-0·94, p=0·021)	0·64 (0·40-1·02, p=0·063)	0·80 (0·55-1·17, p=0·251)	0·78 (0·53-1·15, p=0·202)

OR =Odds Ratio, Data are n (%) unless otherwise stated. ¶ = % of category with positive response BMI = Body Mass Index, IMD = Indices of Multiple Deprivation, WHO = World Health Organisation. Category 3-4 = no continuous supplemental oxygen needed, 5= continuous supplemental oxygen only, 6= Continuous or Bi-level Positive Airway Pressure ventilation or High Flow Nasal O_2, 7-9 = Invasive Mechanical Ventilation or other organ support

Table SR5. Ongoing symptoms recorded at follow-up for the cohort stratified between those with and without pre-existing co-morbidities

	Persisting Symptom	No comorbidity	1+ comorbidity	Total
Total N (%)	··	315 (36·8)	540 (63·2)	855
Any symptom	Yes	201 (86·6)	431 (96·0)	632 (92·8)
Symptom count †	··	7·0 (2·0 to 13·0)	10·0 (5·0 to 17·0)	9·0 (4·0 to 16·0)
Aching in your muscles (pain)	Yes	105 (45·3)	280 (63·5)	385 (57·2)
	(Missing)	83	99	182
Physical slowing down	Yes	95 (41·1)	242 (54·4)	337 (49·9)
	(Missing)	84	95	179
Slowing down in your thinking	Yes	82 (36·0)	201 (45·7)	283 (42·4)
	(Missing)	87	100	187
Joint pain or swelling	Yes	79 (35·3)	236 (54·3)	315 (47·8)
	(Missing)	91	105	196
Limb weakness	Yes	79 (34·3)	231 (52·6)	310 (46·3)
	(Missing)	85	101	186
Difficulty with concentration	Yes	77 (33·9)	191 (43·5)	268 (40·2)
	(Missing)	88	101	189
Short term memory loss	Yes	77 (33·8)	202 (46·2)	279 (42·0)
	(Missing)	87	103	190
Headache	Yes	73 (31·7)	151 (34·2)	224 (33·4)
	(Missing)	85	99	184
Tingling feeling/pins and needles	Yes	60 (26·9)	186 (42·5)	246 (37·2)
	(Missing)	92	102	194
Confusion/fuzzy head	Yes	57 (24·9)	146 (33·0)	203 (30·2)
	(Missing)	86	97	183
Dizziness or lightheaded	Yes	55 (24·7)	163 (37·6)	218 (33·2)
	(Missing)	92	106	198
Chest tightness	Yes	55 (23·7)	126 (28·7)	181 (27·0)
	(Missing)	83	101	184
Problems with balance	Yes	52 (23·3)	183 (41·8)	235 (35·6)
	(Missing)	92	102	194
Altered personality/ behaviour §	Yes	47 (20·3)	93 (21·0)	140 (20·8)
	(Missing)	84	97	181
Chest pain	Yes	42 (18·2)	105 (23·8)	147 (21·9)
	(Missing)	84	99	183
Palpitations	Yes	37 (17·0)	95 (21·8)	132 (20·2)
	(Missing)	97	105	202
Leg/ankle swelling	Yes	39 (16·9)	151 (34·2)	190 (28·3)

	(Missing)	84	99	183
Difficulty with communication	Yes	37 (16·1)	75 (17·0)	112 (16·7)
	(Missing)	85	98	183
Skin rash	Yes	31 (14·0)	73 (17·1)	104 (16·0)
	(Missing)	93	112	205
Diarrhoea	Yes	31 (13·6)	83 (18·7)	114 (17·0)
	(Missing)	87	97	184
Problems seeing	Yes	28 (12·6)	73 (16·7)	101 (15·3)
	(Missing)	93	104	197
Pain on breathing	Yes	26 (11·7)	68 (15·6)	94 (14·3)
	(Missing)	92	105	197
Weight loss	Yes	24 (10·8)	46 (10·6)	70 (10·7)
	(Missing)	92	106	198
Tremor/shakiness	Yes	23 (10·3)	69 (15·8)	92 (13·9)
	(Missing)	92	103	195
Constipation	Yes	21 (9·3)	109 (24·8)	130 (19·5)
	(Missing)	88	101	189
Erectile Dysfunction	Yes	20 (9·2)	75 (17·9)	95 (14·9)
	N/A	72 (33·2)	170 (40·6)	242 (38·1)
	(Missing)	98	121	219
Loss of sense of smell	Yes	21 (9·2)	46 (10·4)	67 (10·0)
	(Missing)	86	97	183
Can't fully move or control movement	Yes	20 (8·9)	54 (12·4)	74 (11·2)
	(Missing)	91	106	197
Abdominal pain	Yes	20 (8·8)	95 (21·4)	115 (17·1)
	(Missing)	87	97	184
Stomach pain	Yes	19 (8·6)	82 (19·0)	101 (15·5)
	(Missing)	94	108	202
Loss of control of passing urine	Yes	19 (8·3)	52 (11·8)	71 (10·6)
	(Missing)	85	98	183
Loss of appetite	Yes	19 (8·2)	68 (15·3)	87 (12·9)
	(Missing)	84	97	181
Loss of taste	Yes	18 (7·8)	52 (11·7)	70 (10·4)
	(Missing)	84	97	181
Nausea/vomiting	Yes	17 (7·5)	51 (11·7)	68 (10·2)
	(Missing)	87	103	190
Bleeding	Yes	13 (6·0)	22 (5·4)	35 (5·6)
	(Missing)	98	129	227
Can't move and/or feel one side of your body or face	Yes	9 (4·0)	30 (6·9)	39 (5·9)

	(Missing)	90	104	194
Loss of control of opening bowels	Yes	7 (3·1)	30 (6·8)	37 (5·5)
	(Missing)	86	100	186
Lumpy lesions on toes	Yes	5 (2·3)	13 (3·2)	18 (2·9)
	(Missing)	100	134	234
Fainting / blackouts	Yes	4 (1·8)	11 (2·5)	15 (2·3)
	(Missing)	93	108	201
Seizures	Yes	2 (0·9)	5 (1·2)	7 (1·1)
	(Missing)	93	106	199

Data are n (%).

Table SR6. Proportion unchanged, worse or better in terms of a) Health-related quality of life (EQ5D-5L) b) Disability (WG-SS) and c) Symptoms at follow-up compared to prior to hospitalisation stratified by severity of acute illness

Table SR6a. EQ5D-5L

	WHO – class 3-4	WHO – class 5	WHO – class 6	WHO – class 7-9	Total
**Do you feel fully recovered from COVID-19? (n - %)*** **	..	..	..	..	..
Yes	51 (30·9)	102 (36·3)	41 (28·5)	45 (18·8)	239 (28·8)
No	75 (45·5)	126 (44·8)	65 (45·1)	163 (67·9)	429 (51·7)
Not sure	39 (23·6)	53 (18·9)	38 (26·4)	32 (13·3)	162 (19·5)
Missing	61	97	41	48	247
**How good or bad is your health overall (EQ5D-5L VAS 0-100)? (Mean -SD)**	..	..	..	..	..
Pre-COVID	78·7 (18·9)	80·0 (17·4)	81·6 (15·7)	84·1 (13·7)	81·1 (16·6)
Post-COVID	70·5 (20·7)	73·8 (18·7)	70·9 (21·4)	69·6 (18·9)	71·5 (19·7)
Change since hospitalisation***	-7·5 (21·6)	-7·9 (17·4)	-9·6 (17·6)	-14·7 (18·9)	-9·9 (19·0)
**EQ5D-5L Utility index (Mean -SD)*** **	..	..	..	..	..
Pre-COVID	0·82 (0·24)	0·84 (0·22)	0·82 (0·23)	0·87 (0·21)	0·84 (0·23)
Post-COVID	0·72 (0·27)	0·76 (0·24)	0·69 (0·29)	0·67 (0·25)	0·71 (0·26)
Change	-0·09 (0·26)	-0·09 (0·20)	-0·11 (0·23)	-0·21 (0·24)	-0·13 (0·24)
**Mobility (n - %)*** **	..	..	..	..	..
No change	103 (67·8)	163 (66·3)	64 (58·2)	95 (49·7)	425 (60·8)
Improvement	14 (9·2)	20 (8·1)	11 (10·0)	6 (3·1)	51 (7·3)
Worse	35 (23·0)	63 (25·6)	35 (31·8)	90 (47·1)	223 (31·9)
(Missing)	74	132	75	97	378
**Self-Care (n - %)** **	..	..	..	..	..
No change	94 (62·7)	156 (63·2)	57 (51·8)	85 (44·5)	392 (56·2)
Improvement	4 (2·7)	3 (1·2)	2 (1·8)	3 (1·6)	12 (1·7)
Worse	52 (34·7)	88 (35·6)	51 (46·4)	103 (53·9)	294 (42·1)
(Missing)	76	131	75	97	379
**Usual Activities (n - %)*** **	..	..	..	..	..
No change	89 (59·3)	156 (63·2)	61 (55·5)	86 (45·0)	392 (56·2)
Improvement	16 (10·7)	14 (5·7)	9 (8·2)	7 (3·7)	46 (6·6)
Worse	45 (30·0)	77 (31·2)	40 (36·4)	98 (51·3)	260 (37·2)
(Missing)	76	131	75	97	379
**Pain/Discomfort (n - %)*** **	..	..	..	..	..
No change	83 (55·3)	143 (58·1)	56 (50·9)	79 (41·4)	361 (51·8)
Improvement	36 (24·0)	53 (21·5)	29 (26·4)	33 (17·3)	151 (21·7)
Worse	31 (20·7)	50 (20·3)	25 (22·7)	79 (41·4)	185 (26·5)
(Missing)	76	132	75	97	380

Anxiety/Depression (n - %)**	..	..	..	..	..
No change	74 (49·7)	142 (57·3)	45 (40·9)	81 (42·4)	342 (49·0)
Improvement	27 (18·1)	23 (9·3)	18 (16·4)	18 (9·4)	86 (12·3)
Worse	48 (32·2)	83 (33·5)	47 (42·7)	92 (48·2)	270 (38·7)
(Missing)	77	130	75	97	379

Missing not included in %, *p<0·05, **p<0·01, ***p<0·0001, column proportions, EQ5D-5L VAS = Euroqol five level visual analogue scale 0-100, WHO = World Health Organisation· Category 3-4 = no continuous supplemental oxygen needed, 5= continuous supplemental oxygen only, 6= Continuous or Bi-level Positive Airway Pressure ventilation or High Flow Nasal O_2, 7-9 = Invasive Mechanical Ventilation or other organ support, WGSS- Washington Group Short Set on Functioning

Table SR6b. Washington Short Set Function score

	WHO –class 3-4	WHO –class 5	WHO –class 6	WHO –class 7-9	Total
**Q1 seeing N (%)**	··	··	··	··	··
No change	130 (81·8)	236 (87·1)	114 (83·2)	194 (83·3)	674 (84·2)
Improvement	2 (1·3)	5 (1·8)	4 (2·9)	3 (1·3)	14 (1·8)
Worse	27 (17·0)	30 (11·1)	19 (13·9)	36 (15·5)	112 (14·0)
Missing N	67	107	48	55	277
**Q2 hearing N (%)**	··	··	··	··	··
No change	143 (90·5)	251 (94·4)	131 (93·6)	198 (86·8)	723 (91·3)
Improvement	3 (1·9)	3 (1·1)	1 (0·7)	4 (1·8)	11 (1·4)
Worse	12 (7·6)	12 (4·5)	8 (5·7)	26 (11·4)	58 (7·3)
Missing N	68	112	45	60	285
**Q 3 walking N (%)*** **	··	··	··	··	··
No change	96 (61·1)	182 (67·7)	88 (64·2)	104 (44·8)	470 (59·1)
Improvement	8 (5·1)	16 (5·9)	7 (5·1)	20 (8·6)	51 (6·4)
Worse	53 (33·8)	71 (26·4)	42 (30·7)	108 (46·6)	274 (34·5)
Missing N	69	109	48	56	282
**Q4 remembering N (%)** **	··	··	··	··	··
No change	96 (60·4)	188 (69·9)	87 (63·0)	119 (51·3)	490 (61·4)
Improvement	6 (3·8)	17 (6·3)	7 (5·1)	11 (4·7)	41 (5·1)
Worse	57 (35·8)	64 (23·8)	44 (31·9)	102 (44·0)	267 (33·5)
Missing N	67	109	47	56	279
**Q5 self-care N (%)*** **	··	··	··	··	··
No change	142 (90·4)	254 (94·4)	125 (89·9)	172 (73·8)	693 (86·8)
Improvement	1 (0·6)	1 (0·4)	1 (0·7)	10 (4·3)	13 (1·6)
Worse	14 (8·9)	14 (5·2)	13 (9·4)	51 (21·9)	92 (11·5)
Missing N	69	109	46	55	279
**Q6 communication N (%)**	··	··	··	··	··
No change	143 (91·1)	249 (93·3)	124 (90·5)	195 (85·5)	711 (90·1)
Improvement	1 (0·6)	4 (1·5)	2 (1·5)	3 (1·3)	10 (1·3)
Worse	13 (8·3)	14 (5·2)	11 (8·0)	30 (13·2)	68 (8·6)
Missing N	69	111	48	60	288
**Any new Disability?** **	41 (25·5)	35 (12·9)	25 (17·9)	57 (24·4)	158 (19·6)

Missing not included in %, column proportions, *p<0·05, **p<0·01, ***p<0·0001, WHO = World Health Organisation· Category 3-4 = no continuous supplemental oxygen needed, 5= continuous supplemental oxygen only, 6= Continuous or Bi-level Positive Airway Pressure ventilation or High Flow Nasal O_2, 7-9 = Invasive Mechanical Ventilation or other organ support

Table SR6c. Symptoms

	WHO – class 3-4	WHO – class 5	WHO – class 6	WHO – class 7-9	Total
**Breathlessness N (%)*** **	..	..	..	..	..
No change	62 (39·7)	115 (44·6)	51 (38·3)	71 (32·3)	299 (39·0)
Improvement	17 (10·9)	37 (14·3)	26 (19·5)	19 (8·6)	99 (12·9)
Worse	77 (49·4)	106 (41·1)	56 (42·1)	130 (59·1)	369 (48·1)
Missing N	*70*	*120*	*52*	*68*	*310*
**Fatigue N (%)** **	..	..	..	..	..
No change	46 (29·7)	97 (37·6)	43 (32·3)	50 (22·9)	236 (30·9)
Improvement	19 (12·3)	40 (15·5)	17 (12·8)	23 (10·6)	99 (13·0)
Worse	90 (58·1)	121 (46·9)	73 (54·9)	145 (66·5)	429 (56·2)
Missing N	*71*	*120*	*52*	*70*	*313*
**Cough N (%)**	..	..	..	..	..
No change	94 (61·0)	170 (66·1)	76 (57·1)	128 (59·0)	468 (61·5)
Improvement	14 (9·1)	35 (13·6)	18 (13·5)	25 (11·5)	92 (12·1)
Worse	46 (29·9)	52 (20·2)	39 (29·3)	64 (29·5)	201 (26·4)
Missing N	*72*	*121*	*52*	*71*	*316*
**Pain N (%)*** **	..	..	..	..	..
No change	76 (50·7)	152 (60·1)	76 (56·7)	81 (37·9)	385 (51·3)
Improvement	16 (10·7)	29 (11·5)	12 (9·0)	18 (8·4)	75 (10·0)
Worse	58 (38·7)	72 (28·5)	46 (34·3)	115 (53·7)	291 (38·7)
Missing N	*76*	*125*	*51*	*74*	*326*
**Sleep N (%)** **	..	..	..	..	..
No change	61 (40·1)	116 (45·3)	60 (44·8)	70 (32·0)	307 (40·3)
Improvement	25 (16·4)	52 (20·3)	22 (16·4)	37 (16·9)	136 (17·9)
Worse	66 (43·4)	88 (34·4)	52 (38·8)	112 (51·1)	318 (41·8)
Missing N	*74*	*122*	*51*	*69*	*316*

Missing not included in %, column proportions. *p<0·05, **p<0·01, ***p<0·0001, WHO = World Health Organisation. Category 3-4 = no continuous supplemental oxygen needed, 5= continuous supplemental oxygen only, 6= Continuous or Bi-level Positive Airway Pressure ventilation or High Flow Nasal O_2, 7-9 = Invasive Mechanical Ventilation or other organ support.

Table SR7. Cluster medoids and characteristics

	Cluster 1	Cluster 2	Cluster 3	Cluster 4
Size	131	159	127	350
**Cluster medoids (z-scores)**	..	..	..	..
Anxiety (GAD-7)	1·5921223	0·5270043	-0·3605941	-0·7156334
Depression (PHQ-9)	1·6495548	-0·066511	-0·222517	-0·846541
PTSD (PCL-5)	1·1880387	0·5344896	-0·3567137	-0·713195
Breathlessness (Dyspnoea-12)	1·2262824	0·3746578	-0·1119849	-0·7202882
Function (FACIT)	1·3974373	0·7784612	-0·4594909	-0·8463509
Physical performance (SPPB)	0·4405809	0·0101025	0·0101025	-0·4203759
Cognition (MoCA)	-0·0714386	-0·6086291	1·2715376	-0·3400339
**Cluster characteristics**	..	..	..	..
Maximal dissimilarity	5·608549	4·146695	6·032896	3·159184
Average dissimilarity	2·539763	1·831245	1·864718	1·10634
Isolation	2·272929	1·680496	3·037516	1·590624

GAD-7 = General Anxiety Disorder 7 Questionnaire, PHQ-9 = Patient Health Questionnaire-9, PCL-5 = Post Traumatic Stress Disorder Checklist, Dyspnoea-12 Questionnaire, FACIT Fatigue Scale (Facit), BPI =Brief Pain Inventory, SPPB = Short Physical Performance Battery, MoCA = Montreal Cognitive Assessment.

Table SR8. Change in primary outcome measures including health-related quality of life and disability after COVID-19 stratified by four recovery clusters

	Cluster 1 'Very Severe'	Cluster 2 'Severe'	Cluster 3 'Moderated & Cognitive'	Cluster 4 'Mild'	Total
Total N (%)	131 (17·1)	159 (20·7)	127 (16·6)	350 (45·6)	767
**Do you feel fully recovered from COVID-19? *****	··	··	··	··	··
Yes^	3 (2·7)	9 (7·0)	36 (36·4)	114 (42·7)	162 (26·6)
No^	95 (84·1)	86 (66·7)	47 (47·5)	98 (36·7)	326 (53·6)
Not sure^	15 (13·3)	34 (26·4)	16 (16·2)	55 (20·6)	120 (19·7)
Missing	18	30	28	83	159
**How good or bad is your health overall (EQ5D-5L VAS 0-100)?**	··	··	··	··	··
Pre-COVID†† ***	74 (18)	80 (15)	84 (15)	86 (13)	81 (17)
Post-COVID †	54 (20)	69 (16)	76 (17)	81 (15)	72 (20)
**EQ5D-5L Utility Index (UI)**	··	··	··	··	··
EQ5D-5L UI Pre-COVID ¥***	0·67 (0·30)	0·83 (0·19)	0·87 (0·17)	0·92 (0·13)	0·84 (0·23)
EQ5D-5L UI Post-COVID ††***	0·43 (0·27)	0·68 (0·17)	0·76 (0·18)	0·87 (0·14)	0·71 (0·26)
Change EQ5D-5L UI††***	-0·25 (0·36)	-0·17 (0·20)	-0·10 (0·18)	-0·05 (0·15)	-0·11 (0·22)
**EQ5D Mobility*****	··	··	··	··	··
No change	32 (40·0)	49 (47·6)	54 (65·9)	195 (78·0)	425 (60·8)
Improvement	5 (6·2)	3 (2·9)	9 (11·0)	15 (6·0)	51 (7·3)
Worse	43 (53·8)	51 (49·5)	19 (23·2)	40 (16·0)	223 (31·9)
Self-Care***					
No change	20 (25·0)	41 (39·4)	54 (65·9)	192 (77·1)	392 (56·2)
Improvement	3 (3·8)	1 (1·0)	1 (1·2)	2 (0·8)	12 (1·7)
Worse	57 (71·2)	62 (59·6)	27 (32·9)	55 (22·1)	294 (42·1)
**Usual Activities*****	··	··	··	··	··
No change	22 (27·5)	47 (45·2)	54 (65·9)	190 (76·3)	392 (56·2)
Improvement	8 (10·0)	3 (2·9)	4 (4·9)	12 (4·8)	46 (6·6)
Worse	50 (62·5)	54 (51·9)	24 (29·3)	47 (18·9)	260 (37·2)
**Pain/ Discomfort*****	··	··	··	··	··
No change	33 (41·2)	45 (43·3)	40 (49·4)	166 (66·7)	361 (51·8)
Improvement	14 (17·5)	23 (22·1)	23 (28·4)	48 (19·3)	151 (21·7)
Worse	33 (41·2)	36 (34·6)	18 (22·2)	35 (14·1)	185 (26·5)
**Anxiety/Depression *****	··	··	··	··	··
No change	9 (11·2)	37 (35·9)	44 (53·7)	175 (70·0)	342 (49·0)
Improvement	20 (25·0)	13 (12·6)	11 (13·4)	25 (10·0)	86 (12·3)
Worse	51 (63·8)	53 (51·5)	27 (32·9)	50 (20·0)	270 (38·7)
'alot of difficulty'	63 (55·8)	33 (26·2)	15 (15·0)	18 (6·8)	204 (24·7)

***§					
‘new disability’***§	57 (51·8)	25 (20·0)	11 (11·5)	12 (4·6)	158 (19·6)

*p<0·05, **p<0·01, ***p<0·0001. Number (%) unless †median [IQR] or ††mean [SD], ¶ = % of category with positive response, § Washington Group Short Set Functioning (WGSS) – ‘a lot of difficulty’ or ‘cannot do at all’ score for any of the seven problems, ‘new disability’ a new score of ‘a lot of difficulty or ‘cannot do at all’ persisting after COVID-19, ^ % calculated after exclusion of missing individuals, EQ5D-5L VAS = Visual Analogue Scale, EQ5D-5L UI = Utility Index

Figure SR1. Forest plot of the patient and admission characteristics associated with recovery using multi-variable logistic regression and multiple imputation with further analysis adjusted for discharge to review time

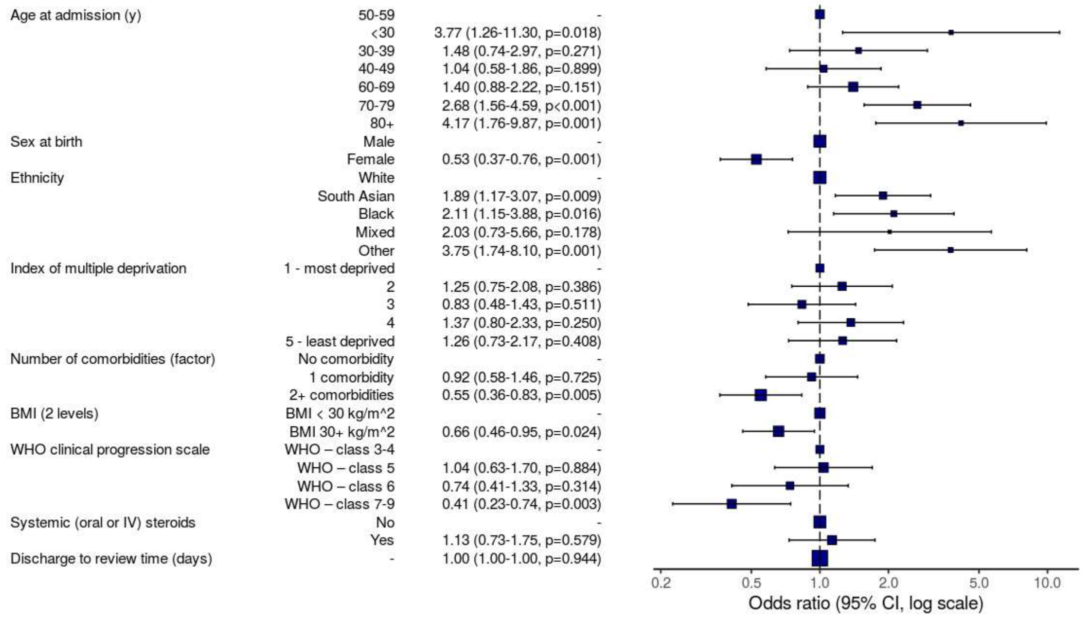

Figure SR2. Histogram of number of symptoms reported at five months after discharge in survivors of a hospital admission due to COVID-19

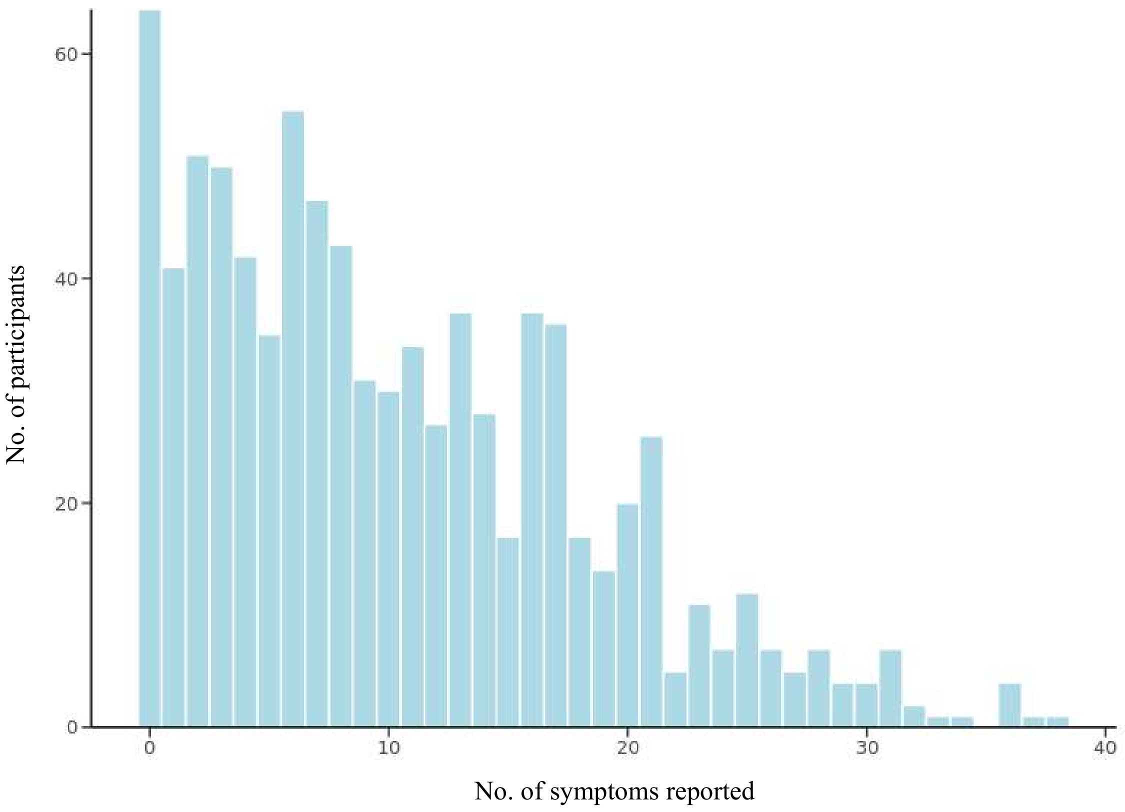

Figure SR3. Health-related quality of life measured by the EQ5D-5L at follow-up compared to prior to admission with COVID-19

EQ5D VAS = visual analogue scale , A) Change in EQ5D-5L domains for whole cohort, B) Change in EQ5D-5L summary metrics for whole cohort, C) Change in EQ5D-5L domains stratified by WHO class of the severity of the acute illness, D) Change in EQ5D-5L summary metrics stratified by WHO class of the severity of the acute illness.

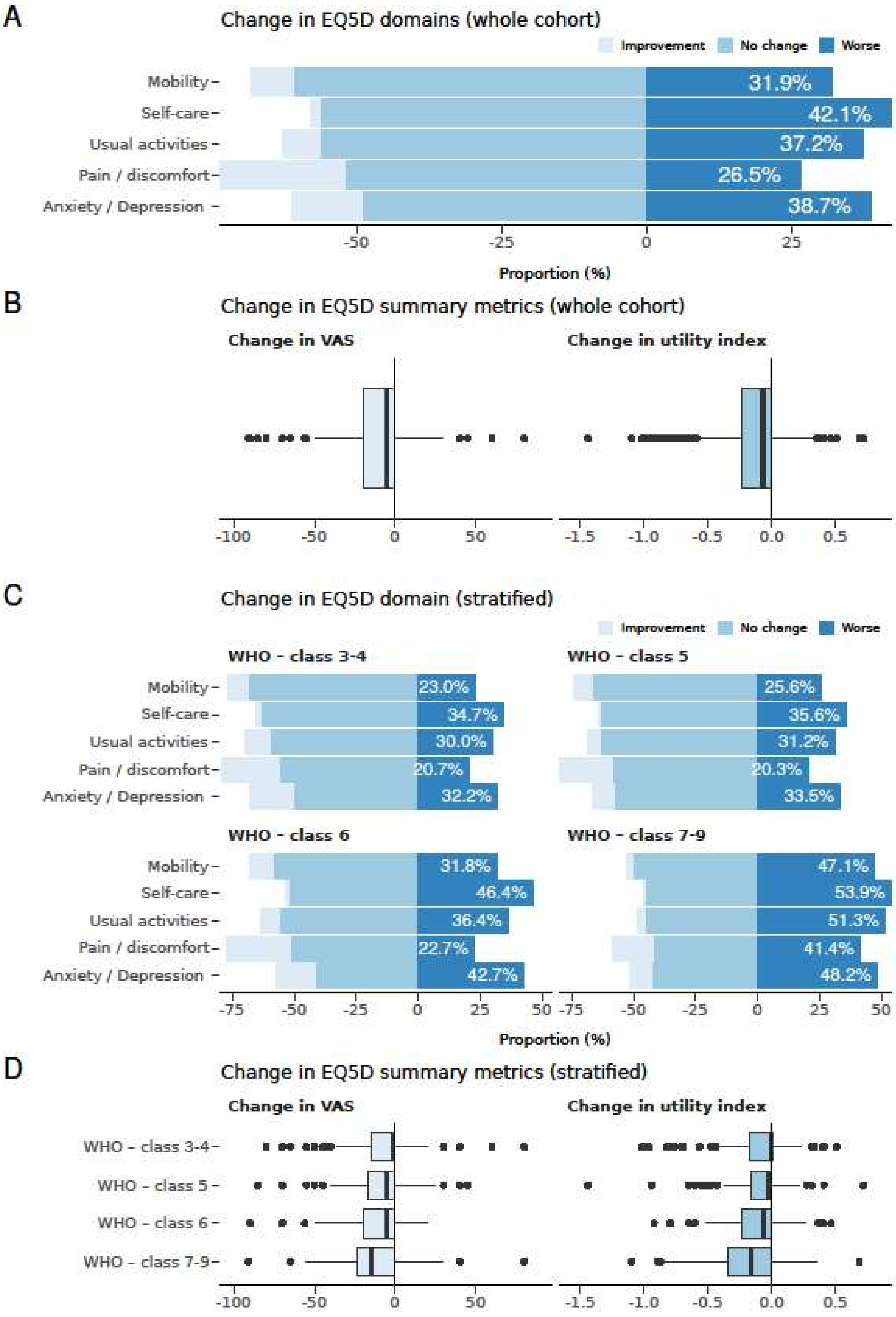

Figure SR4. A comparison between the patient estimated EQ5D Visual Analogue Scale before hospital admission and population values[31]

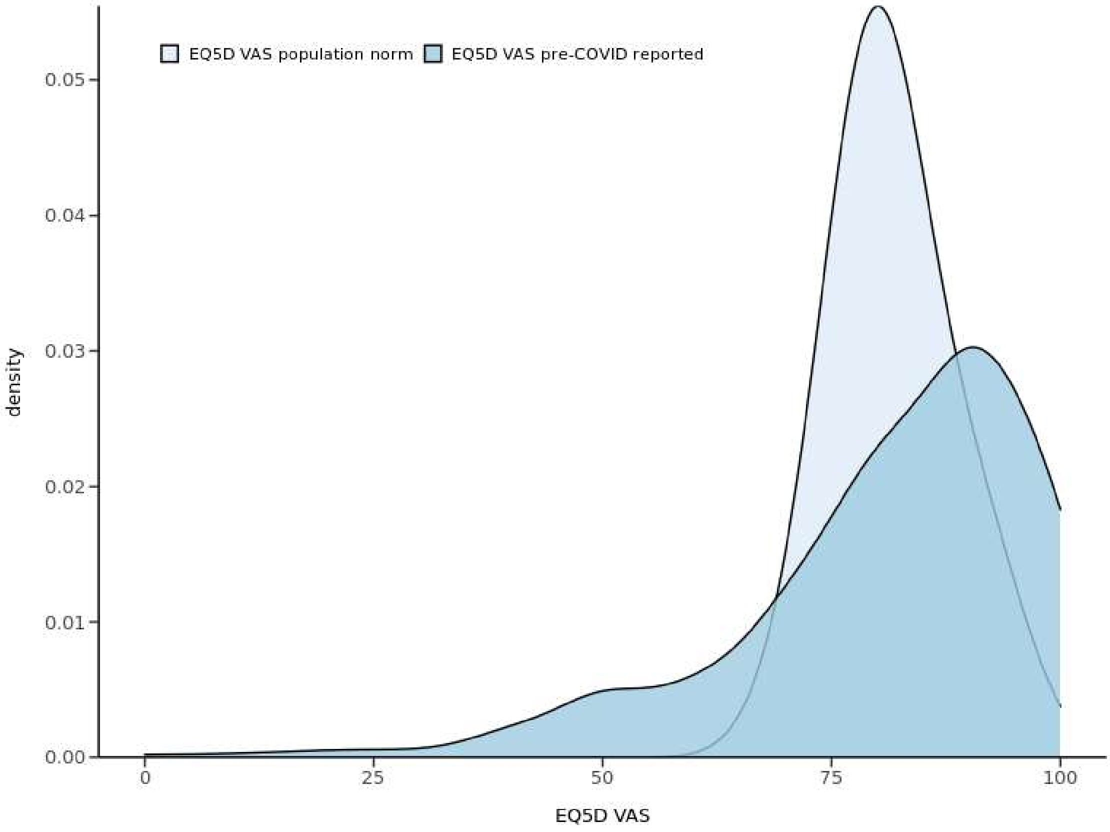

Figure SR5. Clusters of mental, cognitive and physical health impairments

a) Scatter plots for anxiety versus other symptoms, cognition and physical function

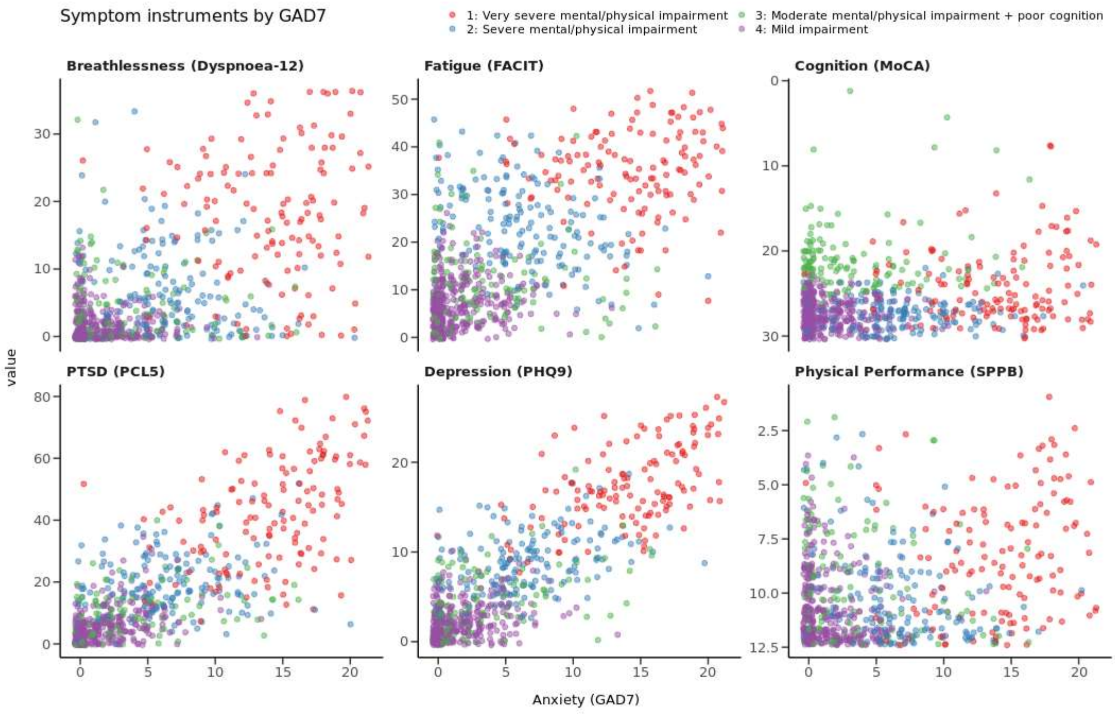

b) Scatter plots for breathlessness versus other symptoms, cognition and physical function

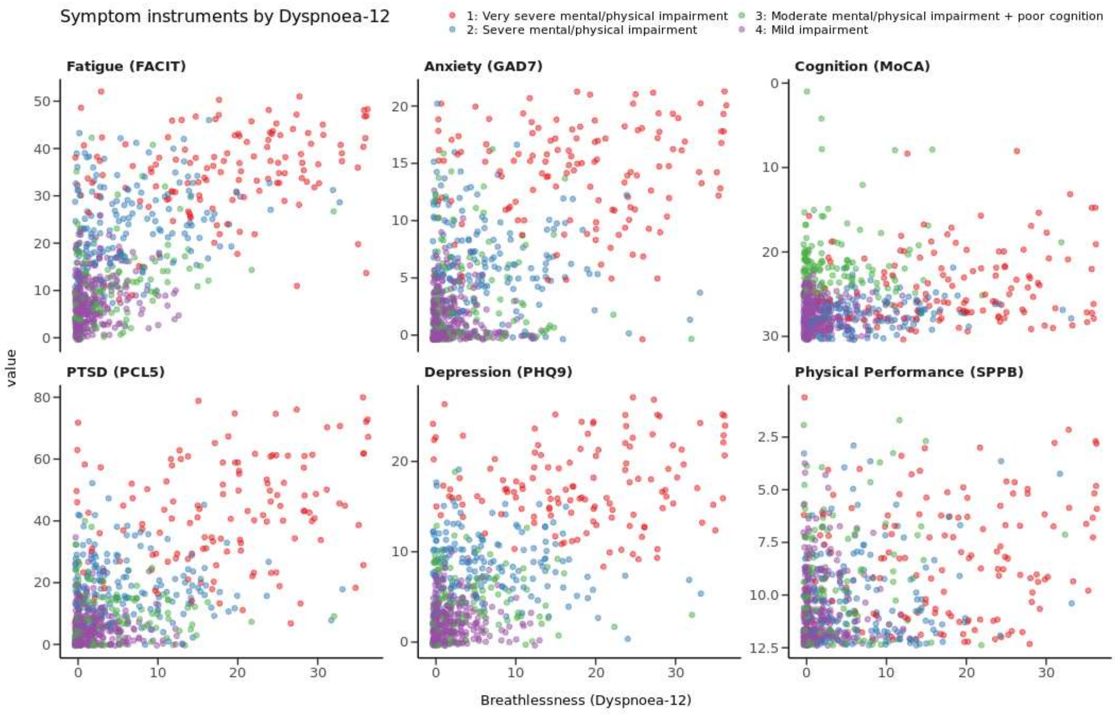

c) Scatter plots for physical function versus other symptoms and cognition

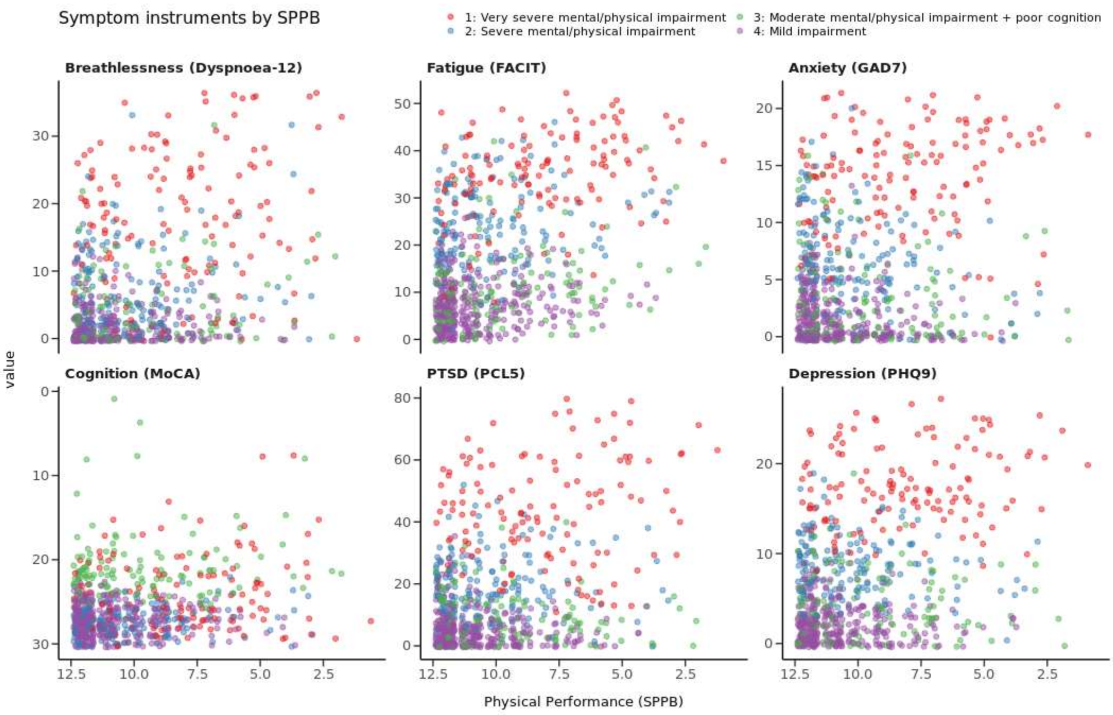

References

1. Office for National Statistics. National Statistics Postcode Lookup (February 2020). February 02, 2020. https://geoportal.statistics.gov.uk/datasets/national-statistics-postcode-lookup-february-2020 (accessed March 01, 2021).
2. Johnson SU, Ulvenes PG, Øktedalen T, Hoffart A. Psychometric properties of the general anxiety disorder 7-item (GAD-7) scale in a heterogeneous psychiatric sample. *Front Psychol* 2019; **10**: 1713.
3. Levis B, Benedetti A, Thombs BD. Accuracy of Patient Health Questionnaire-9 (PHQ-9) for screening to detect major depression: individual participant data meta-analysis. *BMJ* 2019; **365**: l1476.
4. Contractor AA, Elhai JD, Fine TH, et al. Latent profile analyses of posttraumatic stress disorder, depression and generalized anxiety disorder symptoms in trauma-exposed soldiers. *J Psychiatr Res* 2015; **68**: 19-26.
5. Weathers FWL, B.T. ; Keane, T.M. ; Palmieri, P.A. ; Marx, B.P. & Schnurr, P.P. The PTSD Checklist for DSM-5 (PCL-5). 2013. www.ptsd.va.gov (accessed March 01, 2021).
6. Yorke J, Moosavi SH, Shuldham C, Jones PW. Quantification of dyspnoea using descriptors: development and initial testing of the Dyspnoea-12. *Thorax* 2010; **65**(1): 21-6.
7. FACIT.org. Functional Assessment of Chronic Illness Therapy – Fatigue: A 13-item FACIT Fatigue Scale. https://www.facit.org/measures/FACIT-F (accessed March 19, 2021).
8. Butt Z, Lai JS, Rao D, Heinemann AW, Bill A, Cella D. Measurement of fatigue in cancer, stroke, and HIV using the Functional Assessment of Chronic Illness Therapy - Fatigue (FACIT-F) scale. *J Psychosom Res* 2013; **74**(1): 64-8.
9. Cleeland CS. The Brief Pain Inventory User Guide. 2009. https://www.mdanderson.org/documents/Departments-and-Divisions/Symptom-Research/BPI_UserGuide.pdf (accessed March 01, 2021).
10. Cleeland CS, Ryan KM. Pain assessment: global use of the Brief Pain Inventory. *Ann Acad Med Singapore* 1994; **23**(2): 129-38.
11. Vasunilashorn S, Coppin AK, Patel KV, et al. Use of the Short Physical Performance Battery Score to predict loss of ability to walk 400 meters: analysis from the InCHIANTI study. *J Gerontol A Biol Sci Med Sci* 2009; **64**(2): 223-9.
12. Guralnik JM, Simonsick EM, Ferrucci L, et al. A short physical performance battery assessing lower extremity function: association with self-reported disability and prediction of mortality and nursing home admission. *J Gerontol* 1994; **49**(2): M85-M94.
13. Fish J. Short Physical Performance Battery. In: Kreutzer JS, DeLuca J, Caplan B, eds. Encyclopedia of Clinical Neuropsychology. New York, NY: Springer New York; 2011: 2289-91.
14. Singh SJ, Morgan M, Scott S, Walters D, Hardman AE. Development of a shuttle walking test of disability in patients with chronic airways obstruction. *Thorax* 1992; **47**(12): 1019-24.
15. Probst VS, Hernandes NA, Teixeira DC, et al. Reference values for the incremental shuttle walking test. *Respir Med* 2012; **106**(2): 243-8.
16. Rockwood K, Song X, MacKnight C, et al. A global clinical measure of fitness and frailty in elderly people. *CMAJ* 2005; **173**(5): 489-95.
17. Nasreddine ZS. MoCA Montreal Cognitive Assessment Training & Certification. https://www.mocatest.org/training-certification/ (accessed March 01, 2021).
18. Carson N, Leach L, Murphy KJ. A re-examination of Montreal Cognitive Assessment (MoCA) cutoff scores. *Int J Geriatr Psychiatry* 2018; **33**(2): 379-88.
19. Graham BL, Steenbruggen I, Miller MR, et al. Standardization of spirometry 2019 update. An official American thoracic Society and European respiratory Society technical statement. *Am J Respir Crit Care Med* 2019; **200**(8): e70-e88.
20. Quanjer PH, Stanojevic S, Cole TJ, et al. Multi-ethnic reference values for spirometry for the 3–95-yr age range: the global lung function 2012 equations. Eur Respiratory Soc; 2012.

21. Stanojevic S, Graham BL, Cooper BG, et al. Official ERS technical standards: Global Lung Function Initiative reference values for the carbon monoxide transfer factor for Caucasians (vol 50, 1700010, 2017). *Eur Respir J* 2020; **56**(4).
22. Stanojevic S, Graham BL, Cooper BG, et al. Official ERS technical standards: Global Lung Function Initiative reference values for the carbon monoxide transfer factor for Caucasians. *Eur Respir J* 2017; **50**(3).
23. Vestbo J, Hurd SS, Agustí AG, et al. Global strategy for the diagnosis, management, and prevention of chronic obstructive pulmonary disease: GOLD executive summary. *Am J Respir Crit Care Med* 2013; **187**(4): 347-65.
24. Acute heart failure: diagnosis and management. National Institute for Health and Care Excellence; 2014.
25. Chronic heart failure in adults: diagnosis and management. National Institute for Health and Care Excellence; 2018.
26. International Expert Committee Report on the Role of the A1C Assay in the Diagnosis of Diabetes. *Diabetes Care* 2009; **32**(7): 1327-34.
27. Stein PD, Hull RD, Patel KC, et al. D-dimer for the exclusion of acute venous thrombosis and pulmonary embolism: a systematic review. *Ann Intern Med* 2004; **140**(8): 589-602.
28. Herdman M, Gudex C, Lloyd A, et al. Development and preliminary testing of the new five-level version of EQ-5D (EQ-5D-5L). *Qual Life Res* 2011; **20**(10): 1727-36.
29. Gerlinger C, Bamber L, Leverkus F, et al. Comparing the EQ-5D-5L utility index based on value sets of different countries: impact on the interpretation of clinical study results. *BMC Res Notes* 2019; **12**(1): 1-6.
30. Creating Domain Specific Disabilty Indicators Using the WG Short Set on Functioning (WG-SS). 2020. https://www.washingtongroup-disability.com/fileadmin/uploads/wg/Documents/WG_Document__5F_-_Analytic_Guidelines_for_the_WG-SS__Other_Domain_Indicators_.pdf (accessed March 10, 2021).
31. Janssen B SA, Cabases J, editors. Population Norms for the EQ-5D. Self-Reported Population Health: An International Perspective based on EQ-5D [Internet]: Dordrecht (NL): Springer; 2014.
